# Mesothelial cells derived extracellular vesicles promote angiogenesis through the transfer of angiopoietin-2

**DOI:** 10.1371/journal.pone.0353115

**Published:** 2026-07-15

**Authors:** Shelly Loewenstein, Fabian Gerstenhaber, Sapir Milshtain, Stav Leibou, Omri Rahamimov, Alison Siegel, Hannah Sherman, Osnat Sher, Guy Lahat

**Affiliations:** 1 Laboratory of Surgical Oncology, Tel-Aviv Sourasky Medical Center, Tel-Aviv, Israel; 2 Division of Surgery, Tel-Aviv Sourasky Medical Center, Tel-Aviv, Israel; 3 Institute of Pathology, Tel-Aviv Sourasky Medical Center, Tel-Aviv, Israel; 4 Gray Faculty of Medical and Health Sciences, Tel-Aviv University, Tel-Aviv, Israel; The University of Texas Health Science Center at Houston, UNITED STATES OF AMERICA

## Abstract

**Background:**

Peritoneal mesothelial cells play a critical role in shaping the peritoneal tumor microenvironment and are increasingly recognized as active regulators of angiogenesis in cancers that metastasize to the peritoneum, including gastrointestinal (GI) and ovarian malignancies. Through complex interactions with tumor and stromal cells, peritoneal mesothelial cells contribute to the establishment of a pre-metastatic niche in the peritoneal cavity. Extracellular vesicles (EVs), nanosized vesicles secreted by most cell types, mediate intercellular communication by transferring bioactive molecules such as proteins, lipids, and nucleic acids. While tumor-derived EVs have been extensively studied in cancer progression, the role of mesothelial cells-derived EVs in regulating endothelial function and angiogenesis remains largely unexplored.

**Methods:**

EVs were isolated from conditioned media of mesothelial cells and characterized before functional assays. Their effects on endothelial cell behavior were assessed using proliferation, migration, and invasion assays, as well as Matrigel-based tube formation and in vivo angiogenesis plug models. A proteome profiler angiogenesis array was used to identify enriched pro-angiogenic mediators. Mechanistic studies were conducted using lentiviral knockdown and overexpression strategies.

**Results:**

Endothelial cells efficiently internalized mesothelial cells-derived EVs, leading to significant increases in proliferation, migration, invasion, and angiogenesis in vitro and in vivo. Proteomic profiling identified 43 angiogenic regulators within mesothelial cells-derived EVs, with angiopoietin-2 (ANG2) emerging as a key effector. Functional analyses demonstrated that mesothelial cells-derived EVs-induced angiogenesis was mediated through ANG2-TIE2 signaling, with subsequent activation of PI3K, Akt, and ERK1/2 pathways. Importantly, inhibition of ANG2 markedly reduced these angiogenic effects.

**Conclusions:**

This work establishes that EVs secreted by mesothelial cells promote angiogenesis by reprogramming endothelial cells through ANG2-dependent signaling. These findings uncover a novel mechanism of mesothelial-endothelial communication and highlight the ANG2 pathway as a promising therapeutic target in advanced GI cancers with peritoneal metastasis.

## 1. Introduction

Peritoneal mesothelial cells form the cellular lining of the peritoneal cavity and play a central role in regulating the peritoneal tumor microenvironment. Increasing evidence suggests that peritoneal mesothelial cells actively contribute to the establishment of a pre-metastatic niche in cancers that disseminate within the peritoneal cavity, including gastrointestinal and ovarian malignancies. The peritoneal cavity is a frequent site of dissemination for these cancers, and current systemic therapies remain largely ineffective in controlling peritoneal metastasis originating from the pancreas, stomach, colon, or ovary. Consequently, once peritoneal spread occurs, it is associated with aggressive tumor progression, rapid clinical decline, and poor survival [1–4]. Following detachment from the primary tumor, cancer cells can enter the peritoneal cavity through peritoneal fluid and adhere to the mesothelial surface, where they interact with peritoneal mesothelial cells and establish secondary lesions [5,6]. Beneath the mesothelial layer lies the peritoneal membrane, which contains fibroblasts, immune cells such as macrophages and mast cells, as well as vascular and lymphatic networks. After attaching to mesothelial cells, tumor cells can penetrate this barrier and invade underlying tissues, including the omentum and bowel serosa. Angiogenesis, the formation of new blood vessels from pre-existing vasculature, is a critical process supporting the growth and survival of both primary and metastatic tumors. During angiogenesis, endothelial cells (ECs) proliferate, migrate, and organize into tubular structures, ultimately enabling neovascularization [7]. In intraperitoneal tumors, which disseminate primarily by direct seeding rather than hematogenous spread, enhanced vascularization of metastatic sites is a key determinant of tumor progression and survival [8]. Angiopoietin-2 (ANG2), a secreted growth factor, plays a central role in vascular remodeling. Through binding to the TIE2 receptor, ANG2 promotes tumor angiogenesis and inflammatory responses, primarily by activating signaling cascades such as PI3K/Akt and ERK [9–14]. Multiple therapeutic agents targeting the ANG-TIE pathway are currently in development as advanced anti-angiogenic strategies for cancer and ocular diseases [15]. ANG2 is abundantly expressed in ECs and a wide range of tumor types [16–19]; however, its expression in mesothelial cells has not been documented. Extracellular vesicles (EVs) are lipid bilayer-enclosed particles secreted by nearly all cell types [20]. EVs serve as carriers of proteins, nucleic acids, and lipids, thereby mediating intercellular communication. Through this mechanism, EVs contribute to cancer progression by regulating processes such as angiogenesis, invasion, proliferation, and metastasis. Their molecular cargo can influence distant microenvironments, establishing pre-metastatic niches that support tumor spread [21–27]. Although evidence is accumulating regarding the role of mesothelial cells-derived EVs in cancer biology, their contribution to angiogenesis remains unexplored [28–31]. In this study, we investigated the functional and molecular effects of EVs secreted by mesothelial cells on ECs, with a specific focus on ANG2. We demonstrate, for the first time, that mesothelial cells- derived EVs activate the ANG2-TIE2 signaling pathway in ECs, promoting angiogenesis both in vitro and in vivo.

## 2. Materials and methods

### 2.1. Cell culture

Human mesothelial cells (Met-5A, CRL9444™) and human endothelial cells (EA.hy926, CRL2922™) were obtained from the American Type Culture Collection (ATCC). Met-5A cells were maintained in M199 medium supplemented with 3.3 nM epidermal growth factor (EGF), 400 nM hydrocortisone, 870 nM zinc-free bovine insulin, and 20 mM HEPES. EA.hy926 cells were grown in Dulbecco’s modified Eagle’s medium (DMEM). Both media were supplemented with 10% heat-inactivated fetal bovine serum (FBS) and 100 U/ml penicillin-streptomycin (Biological Industries, Israel). Cells were cultured at 37°C in a humidified atmosphere containing 5% CO₂. For experiments involving EV treatment, cells were grown in medium containing 10% EV-depleted FBS.

### 2.2. Isolation of mesothelial cells-derived EVs

Small extracellular vesicles (EVs) from Met-5A mesothelial cells (Met-EVs) were isolated by differential ultracentrifugation [31]. Met-5A cells were cultured in 200 ml serum-free medium for 24 h, after which the conditioned medium was harvested. Sequential centrifugation steps were performed: 3,000 × g for 10 min to remove cell debris and large vesicles, followed by 10,000 × g for 30 min to eliminate medium-sized vesicles. The supernatant was then ultracentrifuged at 100,000 × g for 70 min to enrich small EVs. The pellet was washed in PBS and centrifuged again at 100,000 × g for 70 min [32,33]. All steps were performed at 4°C. The resulting EVs were resuspended in PBS and quantified using a NanoDrop ND-1000 spectrophotometer.

### 2.3. Transmission electron microscopy (TEM)

Samples were placed on formvar/carbon-coated grids, negatively stained with 2% aqueous uranyl acetate for 30 seconds, and subsequently visualized using a JEM-1400 Plus transmission electron microscope (JEOL, Japan).

### 2.4. Nanoparticle tracking analysis (NTA)

EV size distribution and concentration were measured with a NanoSight NS300 (NanoSight, UK). EV suspensions were diluted 1:1000 in 0.1 μm filtered PBS. Three 30-second videos were recorded per sample at 25°C, with a frame rate of 30 fps. Particle movement was analyzed using NanoSight software (version 3.1) [31].

### 2.5. Western blotting

Cells and EVs were lysed in RIPA buffer (Merck). Protein lysates (20 μg from cells, 6 μg from EVs) were resolved by SDS-PAGE. The following antibodies were used: CD81 (Santa Cruz Biotechnology, SC-166029), CD63 (Santa Cruz Biotechnology, SC-5275), CD9 (Abcam, ab92726), ANG2 (Abcam, ab155106), PI3K (Abcam, ab86714), p-Akt (Cell Signaling Technology, #9271), p-ERK1/2 (Cell Signaling Technology, #9101), and β-actin (Cell Signaling Technology, #3700). For signaling analyses, endothelial cells were co-cultured with 10 µg/ml EVs for 24 h before lysis. Protein quantification was performed using densitometry (FUSION FX software), normalized to β-actin.

### 2.6. EVs labeling and uptake assay

EVs were labeled with PKH-67 fluorescent dye as described by Hazan-Halevy et al. and by our group [17,31,34]. Internalization was assessed in EA.hy926 cells using confocal microscopy and flow cytometry. Labeled EVs (2 μg) were incubated with 3 × 10⁵ endothelial cells for different time points. Confocal images were acquired using a Zeiss LSM700 microscope, while flow cytometry data were collected with a FACS Canto II (BD Biosciences) and analyzed using FlowJo software [17,31].

### 2.7. Cell proliferation assay

Endothelial cell proliferation was determined using the XTT cell proliferation kit (Biological Industries). EA.hy926 cells (5,000 cells/well) were seeded in 96-well plates and treated with EVs for 24 h, following the manufacturer’s instructions and as we have described previously [17,31].

### 2.8. Migration and invasion assays

Endothelial cell migration and invasion were tested using Transwell chambers (8 μm pore size, BD Biosciences) as we have described previously [17,31]. For migration, 5 × 10⁴ EA.hy926 cells were seeded in serum-free medium in the upper chamber, while the lower chamber contained DMEM with 2% EV-depleted FBS and 10 µg/ml EVs or recombinant human ANG2 (huANG2) (200 ng/ml) (R&D systems). Invasion assays used inserts coated with Matrigel. After 16 h incubation at 37°C, cells that migrated or invaded were fixed, stained, and quantified in three fields per well using ImageJ software.

### 2.9. Tube formation assay

In vitro angiogenesis was assessed on Matrigel-coated 96-well plates. EA.hy926-GFP, shCtrl EA.hy926-GFP or shANG2 EA.hy926-GFP cells (1 × 10⁵ per well) were seeded in serum-free medium or 10% FBS, in the presence of shCtrl-Met-EVs, shANG2-Met-EVs (10 µg/ml), recombinant human ANG2 (200 ng/ml) (R&D systems), or TIE2 inhibitor (5 µM, Abcam ab141270-B). Tube structures were examined after 12–18 h under a fluorescence microscope. Tube numbers were quantified in five random fields per well (magnification, x100).

### 2.10. Matrigel plug assay in mice

All animal experiments were approved by the Institutional Animal Care and Use Committee, Tel Aviv Sourasky Medical Center (protocol #24-8-19). Animal studies were conducted in accordance with ARRIVE guidelines. All mice were randomly assigned to experimental groups. Whenever feasible, investigators were blinded to group allocation during data collection and analysis. Six-week-old male athymic nude mice (Foxn1nu/+) were injected subcutaneously with 500 μl Matrigel containing either mesothelial cells-derived EVs (100 μg) (n = 5), ANG2-knockdown EVs (100 μg) (n = 5), or PBS (control) (n = 3). After 14 days, all mice were euthanized by CO₂ inhalation followed by cervical dislocation. Matrigel plugs were harvested and analyzed for hemoglobin content (Drabkin’s reagent, Sigma-Aldrich) and CD31 expression by immunohistochemistry (IHC) [35]. To alleviate suffering, all mice were monitored for any signs of pain or distress twice a week. Animals that showed 20% or more weight loss were to be immediately euthanized in accordance with the Guidelines for the Care and Use of Laboratory Animals of Tel Aviv Sourasky Medical Center.

### 2.11. Immunohistochemistry (IHC)

Formalin-fixed, paraffin-embedded sections (4 µm) were stained with anti-CD31 antibody (Abcam, ab28364) according to standard protocols [36].

### 2.12. Human angiogenesis protein array

EVs were analyzed using the Human Angiogenesis Antibody Array G1000 (RayBiotech, USA). A total of 50 µg of EV protein was loaded per array. Fluorescence was measured with an InnoScan® microarray scanner. Mean intensity values were background-subtracted and normalized to positive controls.

### 2.13. Lentiviral knockdown of ANG2

Mission shRNA plasmids (pLKO.1-puro-shANG2 and pLKO.1-puro-shControl, Sigma Aldrich) were co-transfected with psPax2 and pCMV-VSV-G into HEK-293T cells using Lipofectamine 2000 (Invitrogen). Viral supernatants were collected, filtered, and used to infect Met-5A or EA.hy926 cells in the presence of polybrene (8 µg/ml). Stable clones were selected with puromycin (0.5 µg/ml). ANG2 knockdown efficiency was confirmed by Western blot.

### 2.14. Statistical analysis

All experiments were performed in triplicate unless stated otherwise. Data were analyzed using GraphPad Prism software. Comparisons between two groups were performed using two-tailed Student’s t-tests, and comparisons among three or more groups used one-way ANOVA followed by Tukey’s post-hoc test. A p-value ≤ 0.05 was considered statistically significant. Results are presented as mean ± standard deviation (SD).

## 3. Results

### 3.1. Isolation and characterization of mesothelial cells-derived EVs

**Fig 1:**
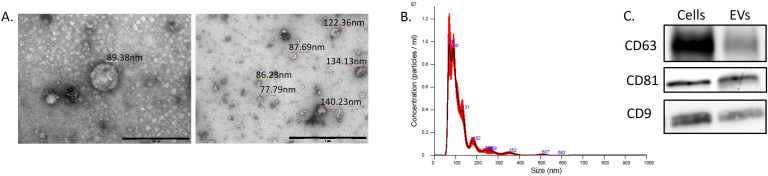
Characterization of mesothelial cells-derived EVs. (A) Transmission electron microscopy (TEM) of EVs isolated from Met-5A cells (Met-EVs). Scale bar represents 200 nm and 1µm (B) Representative image of Nanoparticle tracking analysis (NTA) of isolated Met-EVs (C) Western blot analysis of the EV markers CD63, CD81 and CD9. Full-length blots are presented in Supplementary Figure 1.

To investigate the role of mesothelial cells-derived EVs in angiogenesis, we first isolated small EVs from the conditioned medium of the human mesothelial cell line Met-5A (Met-EVs) using ultracentrifugation as we have previously described [17,31]. The vesicles were characterized by transmission electron microscopy (TEM), nanoparticle tracking analysis (NTA), and Western blotting [31,33,37]. TEM images revealed rounded vesicles ranging from 75–150 nm (Fig 1A). NTA showed a homogeneous population with a modal diameter of 85.3 ± 7.3 nm, consistent with the size of small EVs (<100 nm), and a concentration of 1.34 × 10¹¹ ± 0.32 × 10¹⁰ particles/ml (Fig 1B). Western blot confirmed the expression of the EV markers CD63, CD81, and CD9 in both Met-5A cells and their derived EVs (Fig 1C and S1 Fig). These results confirm successful isolation of small EVs from Met-5A cells.

### 3.2. Uptake of mesothelial cells-derived EVs by endothelial cells

**Fig 2:**
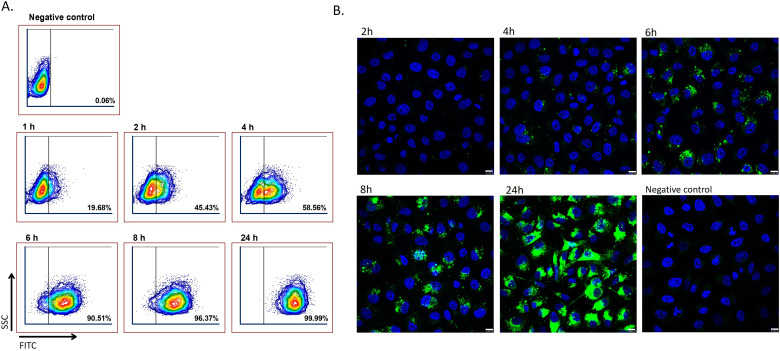
Uptake of mesothelial cells-derived EVs by endothelial cells. (A) Flow cytometry analysis of ECs incubated with PKH-67-labeled Met-EVs. (B) Confocal microscopy confirmed the internalization of labeled EVs within ECs (green: PKH-67 EVs, blue: DAPI nuclei). Negative control: Met-5A cells without labeled EVs. Scale bar represents 10 µm.

To determine whether endothelial cells internalize Met-EVs, fluorescently labeled vesicles were incubated with EA.hy926 transformed human umbilical vein endothelial cells (HUVEC) at different time points. Flow cytometry analysis revealed progressive uptake, with approximately 20% of cells positive for EVs after 1 h, nearly 50% at 2 h, and 96.37% after 8 h (Fig 2A). Confocal microscopy further confirmed internalization of the vesicles by endothelial cells (Fig 2B). These findings indicate efficient and time-dependent uptake of Met-EVs.

### 3.3. Mesothelial cells-derived EVs enhance endothelial cells proliferation, migration, invasion, and tube formation

**Fig 3:**
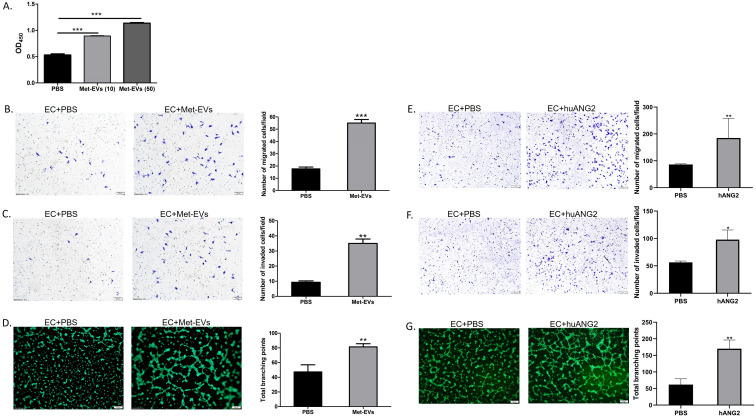
Mesothelial cells-derived EVs promote the proliferation, migration, invasion and tube formation of endothelial cells. (A) Met-EVs increased ECs proliferation. (B,C) Met-EVs enhanced ECs migration and invasion. (D) Met-EVs increased endothelial tube formation of in-vitro. (E, F) Recombinant human ANG2 (huANG2) increased ECs migration and invasion. (G) huANG2 increased endothelial tube formation in-vitro. Representative images (magnification, x100, scale bar represents 50 µm) are shown in the panels. Data are presented as mean ± SD from three independent experiments (n = 3). Comparisons between two groups were performed using two-tailed Student’s t-tests. **p < 0.01; ***p < 0.001.

Next, we evaluated whether Met-EVs influence angiogenic properties of endothelial cells (ECs). ECs co-cultured with Met-EVs exhibited a dose-dependent increase in proliferation: a 67% rise with 10 µg/ml EVs and a 113% rise with 50 µg/ml EVs compared to untreated controls (p < 0.0001; Fig 3A). In migration and invasion assays, Met-EVs enhanced endothelial cell migration 3.0-fold (p < 0.0001) and invasion 3.8-fold (p = 0.001) (Figs 3B, 3C). Additionally, endothelial tube formation increased 1.7-fold following EV treatment (p = 0.004; Fig 3D). To further substantiate the sufficiency of ANG2 in promoting angiogenesis, we repeated the functional assays using recombinant human ANG2 (huANG2). Treatment with huANG2 (200 ng/ml) significantly enhanced ECs migration and invasion by 2.14-fold (p = 0.007) and 1.8-fold (p = 0.01), respectively (Figs 3E, 3F). In addition, huANG2 treatment increased endothelial tube formation by 2.83-fold compared with controls (p = 0.004; Fig 3G). Collectively, these results support a direct pro-angiogenic role for ANG2 and demonstrate that Met-EVs stimulate multiple pro-angiogenic behaviors in ECs.

### 3.4. Mesothelial Cells-Derived EVs carry a broad range of angiogenesis-related proteins

**Fig 4:**
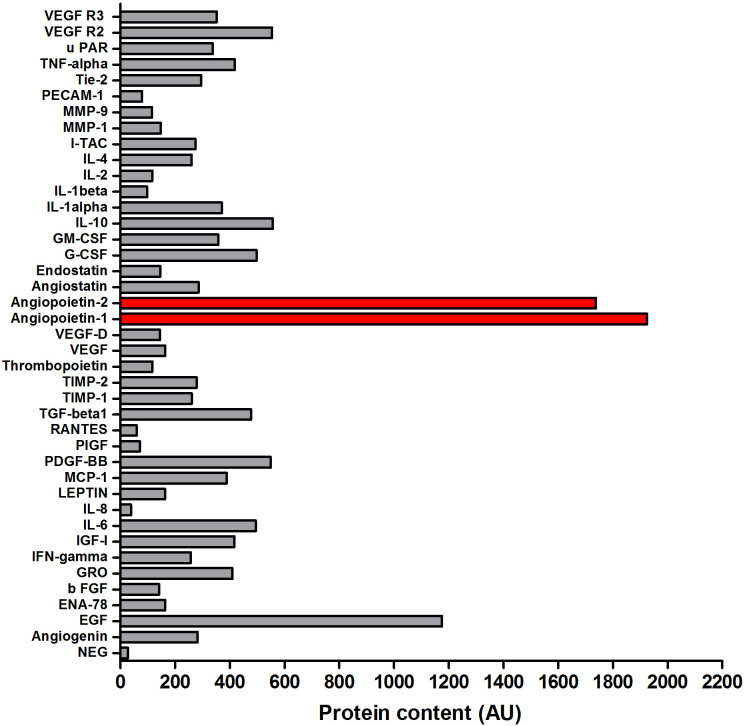
Mesothelial cells-derived EVs contain various angiogenic proteins. A human angiogenesis protein array identified 43 angiogenic proteins within Met-EVs. Signal intensities are shown in arbitrary units (AU).

To identify angiogenic mediators present in Met-EVs, we performed a human angiogenesis protein array. A total of 43 angiogenic proteins were detected, with angiopoietin-1 (ANG1) and angiopoietin-2 (ANG2) being most abundant (1924 and 1738 arbitrary units, respectively) (Fig 4). Additional highly expressed factors included IL-6, IL-10, and TGF-β1 (495, 557, and 478 AU, respectively), all previously linked to peritoneal metastasis [38–40]. Elevated expression of EGF (1175 AU), associated with gastric and ovarian cancer spread, was also observed [41,42]. CXCL1 (410 AU) and PDGF-BB (550 AU), both implicated in peritoneal carcinomatosis, were also enriched [43–45].

These data indicate that Met-EVs contain multiple pro-angiogenic proteins, with ANG2 emerging as a major candidate mediator.

### 3.5. ANG2 from mesothelial cells-derived EVs Activates the Tie2-ANG signaling pathway in endothelial cells

**Fig 5:**
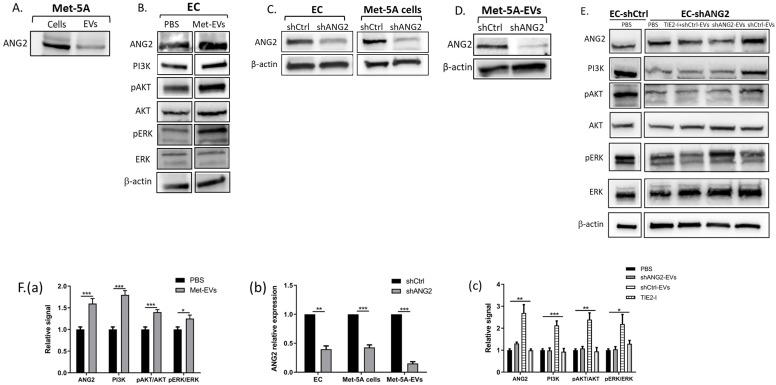
ANG2 from mesothelial cells-derived EVs activates TIE2 signaling and downstream pathways in endothelial cells. (A) Western blot analysis of ANG2 expression in Met-5A cells and their EVs. (B) ECs treated with mesothelial cells-derived EVs (Met-EVs) showed increased ANG2, PI3K, p-Akt, and p-ERK1/2 expression. (C,D) shRNA-mediated ANG2 knockdown reduced ANG2 expression in ECs, mesothelial cells and EVs. (E) ANG2 deficient ECs treated with ANG2-deficient EVs showed reduced activation of PI3K/Akt and ERK signaling; rescue with ANG2-positive EVs restored pathway activation, while TIE2 inhibition blocked this effect. (F) Quantification of protein expression levels normalized to β-actin of western blots from B (a), C – D (b) and E (c). Data are presented as mean ± SD of four independent experiments (n = 4). Comparisons between two groups were performed using two-tailed Student’s t-tests. *p < 0.05; **p < 0.01; ***p < 0.001. Full-length blots are presented in Supplementary Figure 1.

Western blot analysis confirmed that ANG2 was expressed in both Met-5A cells and their secreted EVs (Fig 5A and S1 Fig). The effect of Met-EVs on the expression of ANG2 and three of its downstream proteins: PI3K, p-Akt and pERK1/2, in naïve ECs was evaluated using western blotting. ECs treated with Met-EVs displayed increased expression of ANG2 and downstream signaling proteins PI3K, p-Akt, and p-ERK1/2 (Fig 5B and S1 Fig). To specifically evaluate the role of ANG2, we generated ANG2-knockdown mesothelial cells and ECs using shRNA. Efficient reduction of ANG2 was confirmed in both cell lines (Fig 5C and S1 Fig). Subsequently, lower expression levels of ANG2 were detected in EVs from knockdown mesothelial cells (shANG2-EVs) compared to the control EVs (shCtrl-EVs; Fig 5D and S1 Fig). ECs lacking ANG2 (EC-shANG2) and treated with ANG2-deficient EVs (shANG2-EVs) showed markedly reduced expression of ANG2, PI3K, p-Akt, and p-ERK1/2 compared to control ECs (EC-shCtrl) (Fig 5E and S1 Fig). Importantly, treatment of ANG2-knockdown ECs with control EVs (shCtrl-EVs) restored activation of these pathways, while inhibition of TIE2 (TIE2-I) signaling abrogated this effect (Figs 5E, 5F). Together, these findings indicate that ANG2 delivered by Met-EVs activates the PI3K/Akt and ERK pathways in ECs via TIE2 signaling.

### 3.6. ANG2 in mesothelial cells-derived EVs induces tube formation *In Vitro* and angiogenesis *In Vivo*

**Fig 6:**
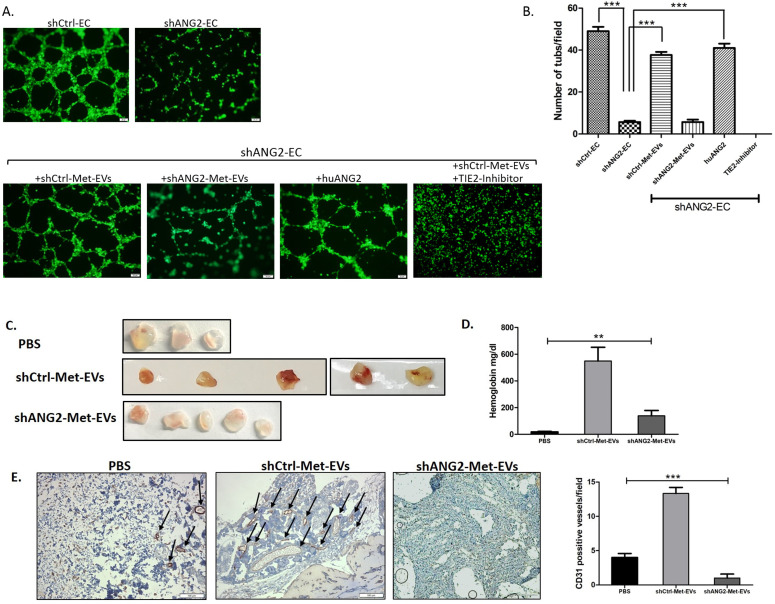
ANG2 delivered by mesothelial cells-derived EVs promotes tube formation in vitro and angiogenesis in vivo. (A) ANG2 knockdown reduced ECs tube formation, whereas treatment with shCtrl-Met-EVs or recombinant human ANG2 (huANG2) restored tube-forming capacity. TIE2 inhibition blocked the effect of ANG2-positive EVs (magnification, x100, Scale bar represents 20 µm). (B) Quantification of tube formation. The data are presented as mean ± SD of four independent experiments (n = 4) (C) Representative Matrigel plugs harvested after 14 days. (D) Hemoglobin content in Matrigel plugs. (E) CD31 immunohistochemistry staining and quantification of blood vessels in Matrigel plugs. Matrigel plug assay: n = 5 for shCtrl-Met-EVs and shANG2-Met-EVs; n = 3 for PBS. Comparisons between two groups were performed using two-tailed Student’s t-tests. Comparisons among three groups used one-way ANOVA followed by Tukey’s post-hoc test. **p < 0.01; ***p < 0.001.

We next investigated the functional impact of Met-EVs-derived ANG2 on angiogenesis. ANG2-deficient ECs (shANG2-EC) displayed a 10-fold reduction in tube-forming capacity compared to control cells (p < 0.0001; Fig 6A, upper panel). When these cells were treated with ANG2-positive EVs (shCtrl-Met-EVs) or recombinant human ANG2 (huANG2), tube formation was restored 6.6-fold and 7.2-fold, respectively (p < 0.0001). Conversely, TIE2 inhibition abolished the ability of ANG2-positive EVs to enhance tube formation (Fig 6A, lower panel; Fig 6B).

In vivo, Matrigel plugs supplemented with ANG2-positive Met-EVs (shCtrl-Met-EVs) exhibited enhanced vascularization compared to plugs with ANG2-deficient EVs (shANG2-Met-EVs) (Fig 6C). Hemoglobin content was significantly higher in plugs containing ANG2-positive EVs (549 mg/dL) compared to those with ANG2-deficient EVs (168 mg/dL; p < 0.01; Fig 6D). Immunohistochemical staining for CD31 revealed a substantial increase in blood vessel density: 13 CD31-positive vessels/field in the ANG2-positive EV group versus only one vessel/field in the ANG2-deficient group (p < 0.001; Fig 6E).

Taken together, these results demonstrate that Met-EVs induce angiogenesis both in vitro and in vivo through delivery of ANG2 and activation of the TIE2 signaling pathway.

## 4. Discussion

In this study, we demonstrate for the first time that extracellular vesicles (EVs) secreted by mesothelial cells exhibit potent pro-angiogenic activity. Specifically, we show that these vesicles transfer ANG2 to endothelial cells (ECs), thereby stimulating angiogenesis through activation of the TIE2–PI3K/Akt and ERK signaling pathways. These findings reveal a previously unrecognized mechanism of communication between mesothelial cells and ECs that may contribute to the vascularization of peritoneal metastases. The peritoneum is lined by mesothelial cells that rest on a supporting layer populated by fibroblasts, immune cells, and vascular and lymphatic network. The role of peritoneal mesothelial cells in intraperitoneal tumor progression remains a subject of ongoing debate. Traditionally, mesothelial cells were considered to function primarily as a passive physical barrier that limits tumor dissemination until the integrity of the mesothelial layer is compromised. However, accumulating evidence suggests that mesothelial cells actively participate in tumor progression by secreting factors that promote processes such as angiogenesis, tumor adhesion, and invasion [46–51]. These observations suggest that mesothelial cells may play a more dynamic role in shaping the tumor microenvironment than previously appreciated.

The concept of the “pre-metastatic niche” describes the early phase of communication between disseminated tumor cells and distant tissues, during which local changes in the microenvironment prepare the target organ for metastatic colonization. In this process, resident stromal cells are not passive bystanders but instead function as “reactive stroma,” initiating signaling cascades that influence tumor cell engraftment and metastatic spread [52]. Well-recognized examples include cancer-associated fibroblasts (CAFs) and tumor-associated macrophages (TAMs), which interact extensively with malignant cells to promote tumor progression and remodeling of the surrounding tissue [53,54]. Within this dynamic microenvironment, continuous bidirectional communication between stromal and tumor cells enables adaptation to the evolving microenvironmental conditions. While soluble cytokines and chemokines have long been recognized as mediators of these interactions, EVs have recently emerged as critical vehicles for intercellular communication. Our findings support the concept that mesothelial cells function as reactive stromal cells by releasing EVs that stimulate angiogenesis, thereby supporting metastatic tumor growth within the peritoneal niche.

Previous studies have demonstrated that mesothelial cells-derived EVs can enhance tumor invasiveness and modify tumor cell secretory profiles. For example, Serrati et al. reported increased expression of TGF-β1 and uPA/uPAR in colorectal cancer cells exposed to mesothelial cell-derived EVs, while Sugimoto et al. demonstrated that EMMPRIN-containing mesothelial cells-derived EVs promoted invasion of diffuse-type gastric cancer cells [55,56]. Importantly, these studies primarily focused on the effects of mesothelial cells-derived EVs on tumor cells. In contrast, the impact of mesothelial cell-derived EVs on ECs has remained largely unexplored. In the present study, we show that mesothelial cells derived EVs are readily internalized by ECs and significantly enhance endothelial proliferation, migration, invasion, and tube formation both in vitro and in vivo, thereby identifying a previously unrecognized role for mesothelial cells-derived EVs in regulating endothelial angiogenic behavior.

Angiogenesis is essential for the survival and expansion of metastatic tumors, particularly within the peritoneal cavity where dissemination occurs predominantly through direct seeding rather than hematogenous spread. In this context, the establishment of a functional vascular network is critical for sustaining tumor growth and progression. Our proteomic analysis identified ANG2 as the dominant angiogenic mediator within mesothelial cells-derived EVs, consistent with its well-established role in destabilizing existing vessels, promoting endothelial sprouting, and driving tumor-associated neovascularization. Consistent with our previous findings demonstrating that gastric cancer–derived EVs promote endothelial angiogenesis through ANG2 [17], proteomic profiling of Met-EVs identified multiple angiogenesis-related proteins, including ANG1, ANG2, GM-CSF, IL-8, and uPAR. Similarly, Sugimoto et al. reported that mesothelial EVs isolated from gastric cancer ascites contain several pro-angiogenic mediators, including GM-CSF, IL-8, and uPAR. Notably, ANG2 was among the most abundant proteins detected in our analysis. Although ANG2 expression has been described in multiple malignancies [57–60], its expression in mesothelial cells and its transfer to ECs via mesothelial cell-derived EVs have not previously been reported. In contrast to the study by Kalfon et al. [17], which focused on the pro-angiogenic effects of gastric cancer–derived EVs, the present work specifically examines EVs derived from mesothelial cells within the peritoneal microenvironment. While ANG2 was identified as a prominent pro-angiogenic cargo in both contexts, our findings demonstrate for the first time that mesothelial cells-derived EVs themselves can actively promote angiogenesis. These results suggest that both tumor-derived and stromal EVs may cooperate to promote angiogenesis within the peritoneal metastatic niche through delivery of ANG2 to ECs. Following peritoneal invasion, metastatic gastric cancer cells and mesothelial cells may therefore act in concert to release EVs that coordinate angiogenic signaling within the tumor microenvironment.

Mechanistically, ANG2 promotes angiogenesis through binding to the TIE2 receptor and activation of downstream signaling pathways including PI3K/Akt and ERK1/2 [11]. In our study, mesothelial cells-derived EVs enhanced the expression of ANG2, PI3K, p-Akt, and pERK1/2 in ANG2-knockdown ECs. These findings support a model in which EV-delivered ANG2 synergizes with endogenous ANG2 to activate TIE2 signaling in both paracrine and endocrine manners. Functional tube-formation assays further demonstrated that ANG2-containing EVs restore angiogenic capacity in ANG2-deficient ECs, an effect that was abolished following pharmacological inhibition of TIE2. Although TIE2 inhibition may also interfere with signaling mediated by other ligands such as ANG1, these results nonetheless support a central role for the ANG2–TIE2 signaling axis in mediating the pro-angiogenic effects of mesothelial cells-derived EVs in ECs. It should be noted that direct receptor-binding experiments were not performed in the present study, which represents a limitation. Future investigations incorporating co-immunoprecipitation or additional receptor-binding approaches will be important to further confirm this interaction at the molecular level.

From a therapeutic perspective, these findings highlight the ANG–TIE signaling axis as a potential target in metastatic cancers. Current anti-angiogenic strategies primarily focus on inhibition of VEGF signaling; however, the long-term efficacy of these therapies is frequently limited by adaptive resistance mechanisms. Targeting the ANG2/TIE2 pathway therefore represents a promising complementary approach, and several therapeutic agents directed against this signaling axis are currently under clinical investigation.

The ANG–TIE signaling network operates within a complex regulatory framework that includes upstream mediators such as VEGF, with the MAPK/ERK pathway reported to contribute to ANG2 regulation in certain contexts. Although our findings support ERK activation downstream of EV-mediated ANG2–TIE2 signaling in ECs, we did not fully delineate the hierarchical relationship between VEGF signaling, ERK activation, and ANG2 expression. Additional studies will therefore be required to clarify the precise positioning and crosstalk among these pathways in the context of EV-driven angiogenesis. The ANG–TIE signaling pathway is further regulated by multiple molecules, including ANG1, VEGF, and HIF1α, which may function as agonists or antagonists of TIE2 signaling. ANG1 acts as a natural agonist of the TIE2 receptor and promotes vascular stabilization, whereas ANG2 frequently functions as a context-dependent antagonist that contributes to vascular destabilization. Importantly, although ANG2 has been reported to promote endothelial destabilization and apoptosis in the absence of VEGF, its biological activity is highly context-dependent and influenced by the presence of additional pro-angiogenic signals. In our experimental system, despite relatively low VEGF levels, Met-EVs contain additional angiogenic mediators such as ANG1, IL-8, GM-CSF, and uPAR, which may cooperate with ANG2 to promote endothelial activation and angiogenesis. A deeper understanding of the complex interplay among ANG1, ANG2, VEGF, and HIF1α will be critical for the development of effective therapeutic strategies targeting the ANG–TIE pathway [15,59].

In conclusion, our findings identify mesothelial cell-derived EVs as previously unrecognized mediators of angiogenesis within the peritoneal metastatic microenvironment. By transferring ANG2 to endothelial cells, these EVs promote angiogenic signaling and endothelial activation. These insights provide a conceptual framework for the development of therapeutic strategies aimed at disrupting EV-mediated angiogenic communication within the peritoneal niche, which may ultimately contribute to improved treatment outcomes for patients with peritoneal metastases.

## Supporting information

S1 Fig Full length uncropped Western blots for Figure 1 and Figure 5. (Figure 1C) Full length western blots using anti-CD63, anti CD81 and anti CD9. (A) Full length western blot using anti-ANG2. (B) Full length western blot using anti ANG2, anti PI3K, anti pERK, anti ERK, anti pAKT, anti AKT and anti B-Actin antibodies. (C) Full length western blot using antiANG2 and anti B-Actin. (D) Full length western blot using anti ANG2 and anti B-Actin antibodies. (E) Full length western blot using anti ANG2, anti pERK, anti ERK, anti PI3K, anti pAKT, anti AKT and anti B-Actin antibodies.
(PDF)
