# Supplementary figures and images for: Mesothelial cells derived extracellular vesicles promote angiogenesis through the transfer of angiopoietin-2
Source: PLoS One. 2026 Jul 15;21(7):e0353115. doi: 10.1371/journal.pone.0353115 (PMC13372138; doi:10.1371/journal.pone.0353115)

Figure 1C

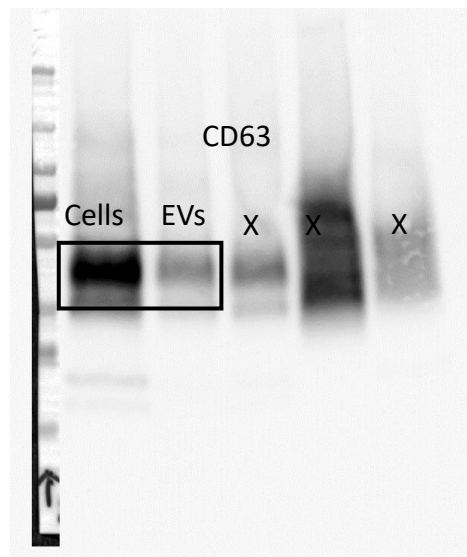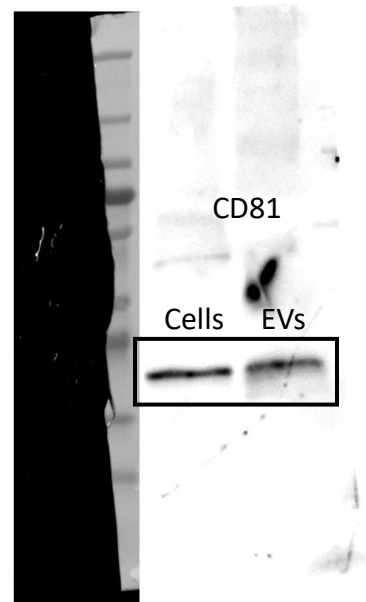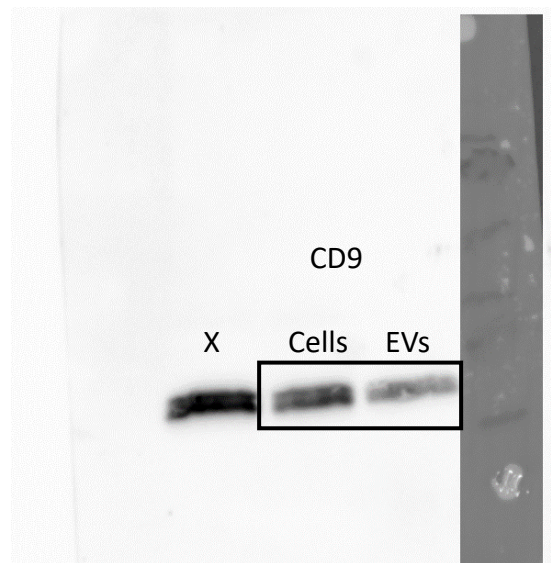

Figure 5A

A.

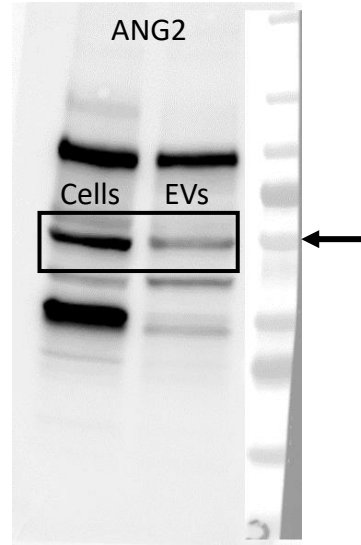

Figure 5B

B.

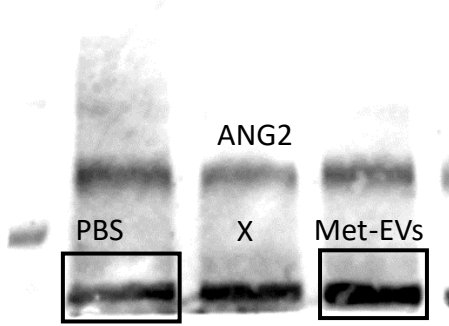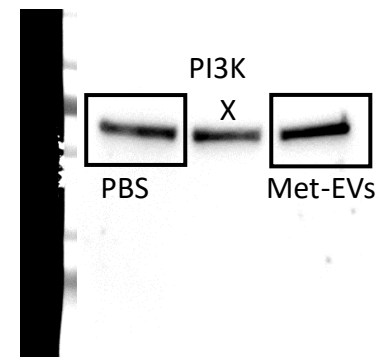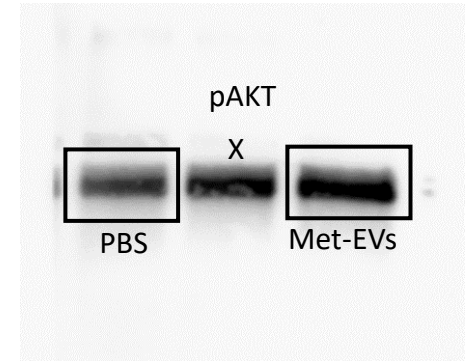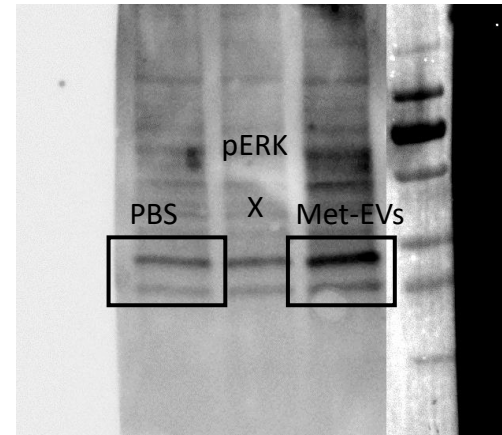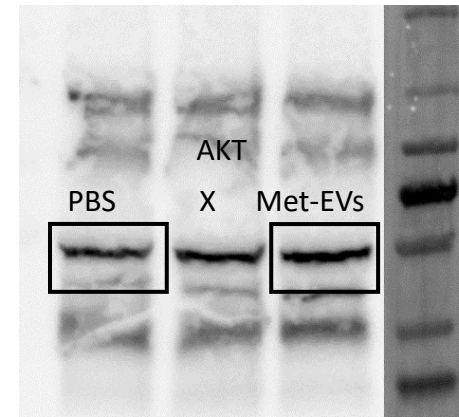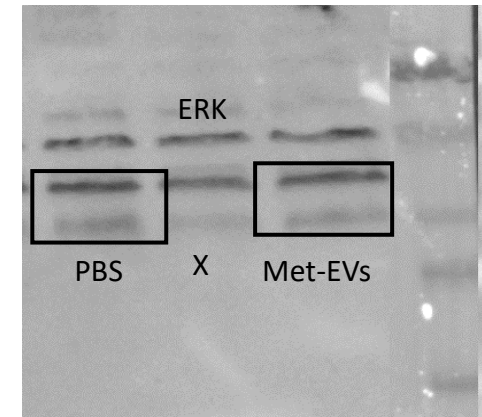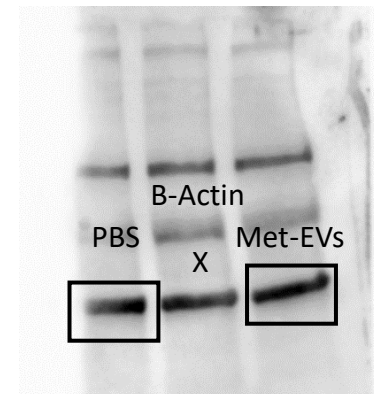

Figure 5C

C.

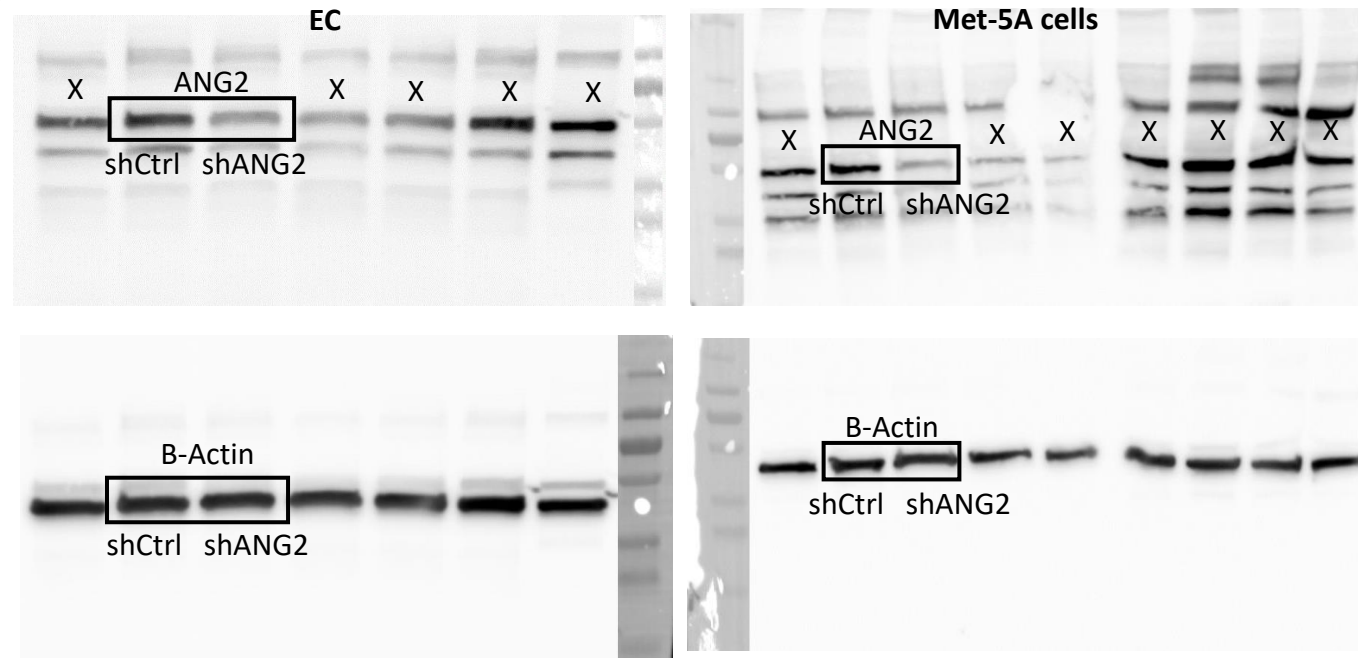

Figure 5D

D.

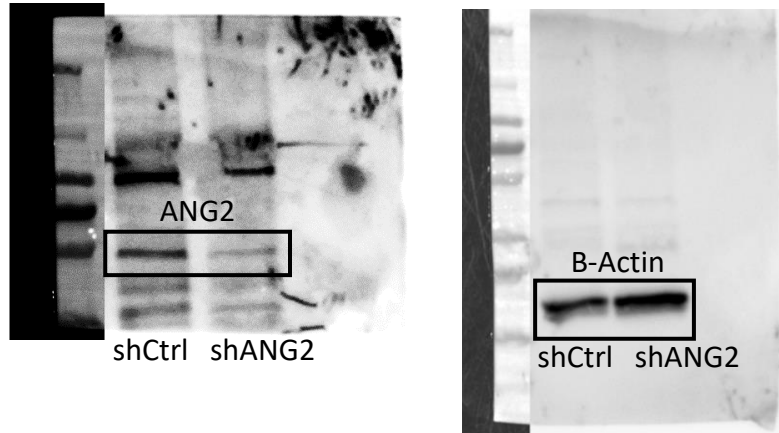

Figure 5E

E.

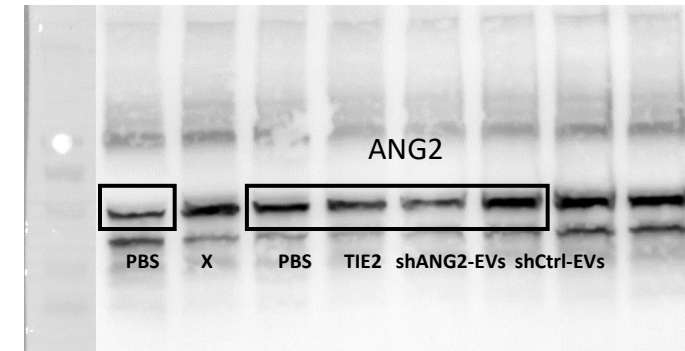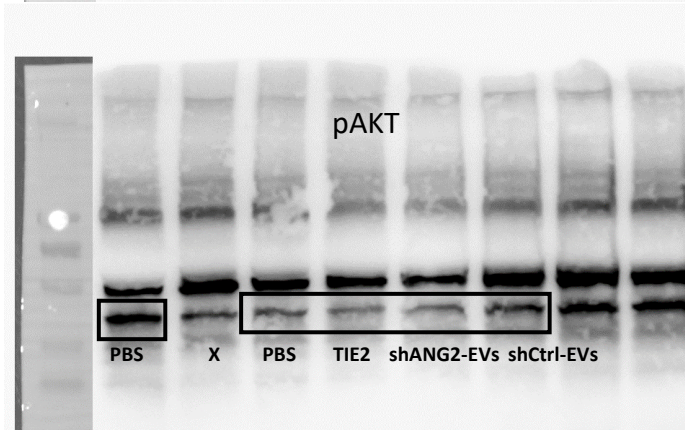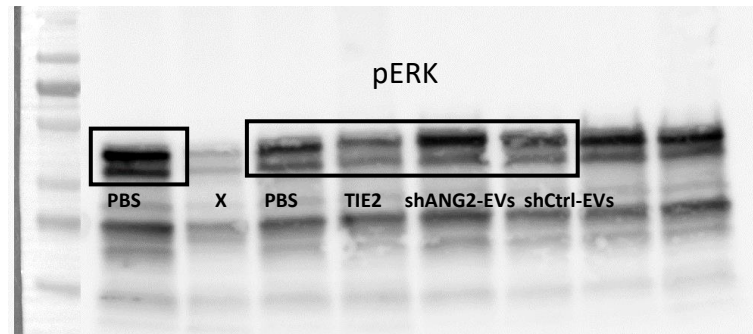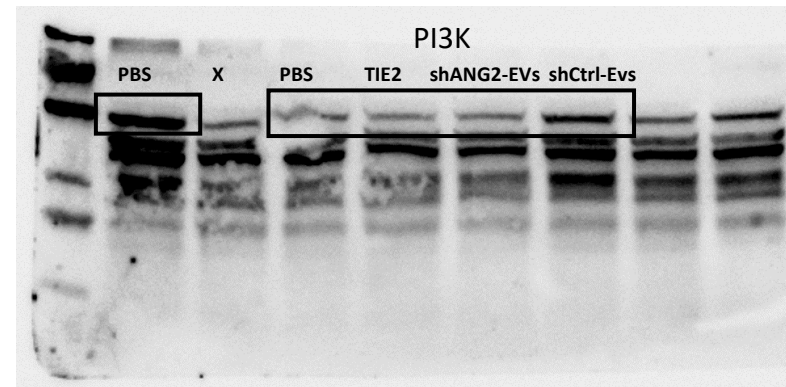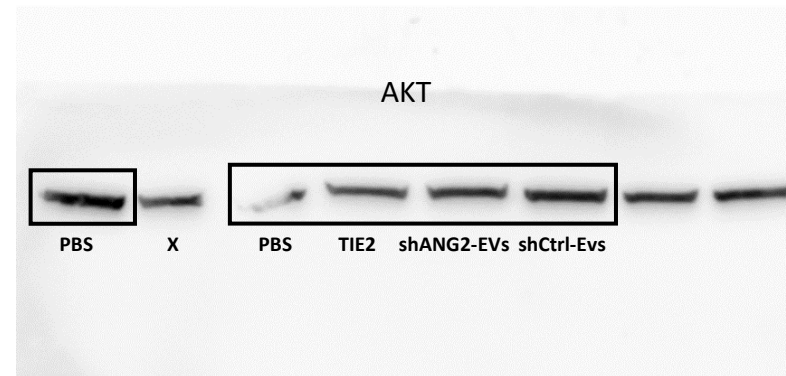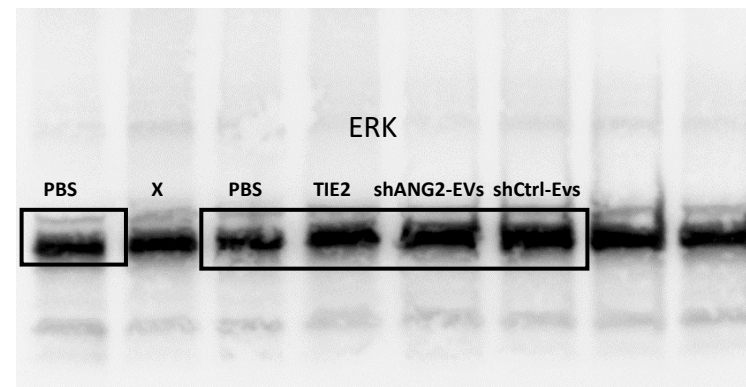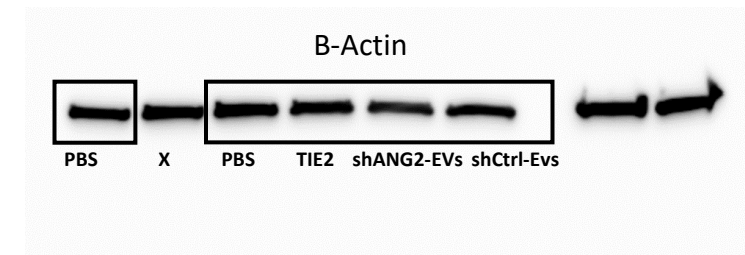

Supplement: S1 Fig — (Figure 1C) Full length western blots using anti-CD63, anti CD81 and anti CD9. (A) Full length western blot using anti-ANG2. (B) Full length western blot using anti ANG2, anti PI3K, anti pERK, anti ERK, anti pAKT, anti AKT and anti B-Actin antibodies. (C) Full length western blot using antiANG2 and anti B-Actin. (D) Full length western blot using anti ANG2 and anti B-Actin antibodies. (E) Full length western blot using anti ANG2, anti pERK, anti ERK, anti PI3K, anti pAKT, anti AKT and anti B-Actin antibodies. (PDF) [file pone.0353115.s001.pdf]
