# Supplementary material for: Mesothelial cells derived extracellular vesicles promote angiogenesis through the transfer of angiopoietin-2
Source: PLoS One. 2026 Jul 15;21(7):e0353115. doi: 10.1371/journal.pone.0353115 (PMC13372138; doi:10.1371/journal.pone.0353115)
Supplement: Attachment — Submitted filename: Response letter-Plos ONE-8.6.26.docx [file pone.0353115.s004.docx]

Dear Dr. Yu A. An,

We would like to thank the reviewers for their careful evaluation of our revised manuscript (PONE-D-25-48703R1) and for their positive comments and recommendation for publication.

We are grateful for the reviewer's final suggestions regarding language polishing, simplification of figure legends, and consistency in statistical reporting. In response, we have carefully reviewed the entire manuscript and implemented the recommended revisions. Specifically, we performed additional language editing to improve clarity and readability, simplified the figure legends where appropriate, and ensured consistency in the presentation and reporting of statistical analyses throughout the manuscript.

We appreciate the reviewers' constructive feedback, which has helped improve the quality of our work. We hope that the revised manuscript is now suitable for publication.

Sincerely,

Shelly Loewenstein, PhD

On behalf of all authors

Reviewer Comment: Minor revisions including final language polishing, simplification of figure legends, and consistency in statistical reporting are recommended.

Response: We thank the reviewer for these suggestions. We have carefully revised the manuscript, accordingly, including additional language polishing, simplification of figure legends, and harmonization of statistical reporting throughout the text, tables, and figures.
