# Supplementary material for: Mesothelial cells derived extracellular vesicles promote angiogenesis through the transfer of angiopoietin-2
Source: PLoS One. 2026 Jul 15;21(7):e0353115. doi: 10.1371/journal.pone.0353115 (PMC13372138; doi:10.1371/journal.pone.0353115)
Supplement: Attachment — Submitted filename: Response letter PlosONE 3.9.25.docx [file pone.0353115.s002.docx]

Dear Dr., Chenette,

Thank you for your careful review of our manuscript and for giving us the opportunity to revise and resubmit.

We acknowledge that there was textual overlap between our submitted manuscript and a previously published paper from our laboratory, particularly in the *Materials and Methods* section (Kalfon et al, 2022). Since the experimental procedures are similar, some degree of similarity was unavoidable. However, we fully understand the importance of ensuring originality and have therefore carefully rephrased and revised all overlapping text throughout the manuscript.

Importantly, the current study presents new data, results, and interpretations that were not included in the previous publication. We believe these findings add substantial novel insights to the field.

We appreciate your consideration and the opportunity to improve our work. We hope that the revised version now meets the journal’s requirements.

Sincerely,

Shelly Loewenstein

On behalf of all co-authors
