# Supplementary material for: Mesothelial cells derived extracellular vesicles promote angiogenesis through the transfer of angiopoietin-2
Source: PLoS One. 2026 Jul 15;21(7):e0353115. doi: 10.1371/journal.pone.0353115 (PMC13372138; doi:10.1371/journal.pone.0353115)
Supplement: Attachment — Submitted filename: Response letter-Plos ONE-15.3.26.docx [file pone.0353115.s003.docx]

Dear Dr. Chenette,

On behalf of my co-authors, I would like to thank you and the reviewers for the careful evaluation of our manuscript entitled “*Mesothelial cells–derived extracellular vesicles promote angiogenesis through the transfer of angiopoietin-2*” by S. Loewenstein et al. (Manuscript ID: PONE-D-25-48703).

We greatly appreciate the constructive and insightful comments provided by the reviewers. These suggestions were extremely helpful and allowed us to improve the clarity, rigor, and overall quality of the manuscript. In response, we have carefully revised the manuscript to address all reviewer comments. Major revisions include clarification of the ANG2–TIE2 signaling mechanism, expansion of the discussion regarding the interplay between ANG1, ANG2, and VEGF, improvement of several figures, and additional methodological clarifications.

Detailed point-by-point responses to the reviewers’ comments are provided below. All corresponding changes in the manuscript have been clearly indicated, including page and paragraph numbers where applicable.

We believe that the revised manuscript provides novel insight into the role of mesothelial cell–derived extracellular vesicles in regulating angiogenesis. The revisions have significantly strengthened the manuscript and address the reviewers’ concerns. We hope that the manuscript will now be suitable for publication in PLOS ONE.

Thank you for your time and consideration.

Sincerely,

Shelly Loewenstein, PhD

(on behalf of all co-authors)

**Reviewer #1**: The manuscript provides strong evidence that mesothelial cell–derived extracellular vesicles (EVs) enhance angiogenesis via ANG2–TIE2 signaling. The study is well-designed, methodologically sound, and relevant to peritoneal metastasis research. Some clarifications and refinements are required before publication.

Major Revisions:
1. Clarify Novelty

Clearly describe how this work differs from Kalfon et al., 2022, specifying new experiments (e.g., in vivo Matrigel plug assay, ANG2 knockdown) and novel mechanistic findings.

We thank the reviewer for this important comment. Kalfon et al. (2022) focused on the pro-angiogenic effects of extracellular vesicles (EVs) derived from tumor cells (i.e., gastric cancer cells). In contrast, the present study investigates the angiogenic effects of EVs derived from cells within the tumor microenvironment and the metastatic peritoneal niche, specifically mesothelial cells. Thus, while the previous work addressed tumor cell–derived EVs, the current study shifts the focus to stromal components and their contribution to angiogenesis. In both studies, ANG2 was identified as one of the most abundant pro-angiogenic proteins present in EVs. However, the biological context and mechanistic implications differ significantly. In the current work, we demonstrate for the first time that mesothelial cell–derived EVs actively promote angiogenesis, highlighting their role in shaping the pre-metastatic niche and facilitating metastatic progression. Moreover, this study extends beyond our prior work by incorporating additional experimental approaches, including the in vivo Matrigel plug assay using the ANG2 knockdown EVs, which provide mechanistic evidence supporting the functional role of mesothelial EV-derived ANG2 in angiogenesis. Together, these findings establish a novel role for mesothelial cell–derived EVs in promoting angiogenesis as part of the contribution of the pre-metastatic niche to metastatic progression, thereby clearly distinguishing this work from Kalfon et al. (2022).

The comment was addressed, and a short paragraph was added to the discussion section (see discussion section, page 15, row 353-362).

2. Mechanistic Evidence

Note that direct receptor-binding validation (e.g., co-IP or blocking assay) was not performed; acknowledge this as a limitation and suggest future confirmation.

Although we did not perform direct receptor-binding validation such as co-immunoprecipitation (co-IP), we did conduct functional blocking experiments using a TIE2 inhibitor (TIE2-I). As shown in Figures 5E and 5F, endothelial cells lacking ANG2 and treated with ANG2-deficient EVs exhibited reduced activation of the PI3K/Akt and ERK signaling pathways. Rescue with ANG2-positive EVs restored pathway activity, whereas inhibition of TIE2 abrogated this effect. (see results section, page 11, rows 274-277 and figure 5E and 5F).

While these findings functionally support the involvement of the ANG2–TIE2 axis, we acknowledge that direct receptor-binding assays were not performed and represent a limitation of the current study. Future studies incorporating co-IP or additional receptor-binding approaches will be important to further validate this interaction at the molecular level.

The comment was addressed, and a short paragraph was added to the discussion section (see discussion section, page 15, row 373-376).

3. Data and Figure Transparency

Improve the resolution and labeling of Figures 3 and 5. Include biological replicates (n), statistical tests used, and exact p-values in figure legends. Specify whether error bars represent SD or SEM.

We thank the reviewer for this helpful comment. Figures 3 and 5 have been revised to improve resolution and labeling. In addition, where information was previously missing, we have updated the figure legends to include the number of biological replicates (n), the statistical tests used, the exact p-values, and a clear specification of whether error bars represent SD or SEM. Revised, higher-resolution versions of Figures 3 and 5 have been incorporated into the manuscript.

4. Animal Study Details

Provide group sample sizes, information on randomization/blinding, and add a statement confirming compliance with ARRIVE guidelines.

We thank the reviewer for this important comment. Group sample sizes are now clearly indicated in the legend of Figure 6 and have also been added to the *Matrigel Plug Assay in Mice* subsection in the Materials and Methods section. We further clarify that all mice were randomly assigned to experimental groups. Whenever feasible, investigators were blinded to group allocation during data collection and analysis. In addition, we have included a statement confirming that all animal experiments were conducted in accordance with ARRIVE guidelines. (see materials and methods section, page 7, row 174-179 and figure 6 legend).

Minor Revisions:

1.Abstract

Mention the identification of 43 angiogenic regulators and emphasize ANG2 as the key mediator. Conclude with a sentence on the therapeutic potential of targeting ANG2.

We thank the reviewer for this thoughtful suggestion. These points are now clearly articulated in the revised abstract to better reflect the scope of our findings and the potential clinical relevance of targeting ANG2.

2. Terminology and Formatting

Ensure consistent use of abbreviations (EVs, Met-EVs, PMCs, ECs) and standardized symbols (µg, µM). Maintain uniform formatting of gene and protein names.

We thank the reviewer for this careful observation. The manuscript has been thoroughly reviewed to ensure consistent use of abbreviations (EVs, Met-EVs, PMCs, ECs) and standardized units (µg, µM) throughout. In addition, gene and protein names have been uniformly formatted according to standard conventions. All necessary corrections have been implemented in the revised version of the manuscript.

3. References

Verify formatting and remove duplicates (especially #15 and #26). Add at least one recent (2023–2024) reference on EV-mediated angiogenesis.

We thank the reviewer for this helpful comment. The reference list has been carefully reviewed to verify formatting consistency. In addition, we have incorporated three recent references (2024–2025) addressing EV-mediated angiogenesis to ensure the manuscript reflects the most up-to-date literature in the field (references 20-22).

4. Language and Style

Shorten long sentences in the Discussion (lines 296–350) for clarity. Maintain a consistent academic tone and correct minor typographical errors.

We thank the reviewer for this helpful comment. The Discussion section has been carefully revised to shorten long sentences and improve overall clarity and readability. We have also ensured a consistent academic tone throughout and corrected minor typographical errors in the revised manuscript.

**Reviewer #2**: The authors present evidence that angiopoietin-2 is present in met-EVs and these EVs are taken up by endothelial cells. The experiments show a necessity of angiopoietin-2 through in vitro assays for cell proliferation, migration, invasion, and tube formation. However, the current manuscript lacks the necessary experiments to mechanistically and physiologically discern the role of angiopoietin-2 in cell signaling and to control for its interplay with angiopoietin-1, VEGF, and milieu of other met-EV factors.

Major Concerns:

1. EC-shCtrl treated with PBS show the strongest signal for all protein and phospho-protein levels. Such a robust signal suggests a nonspecific or artificial activation of the pathway. Furthermore, the signals in 5F do not visually match the 5E blots.

We thank the reviewer for this important comment. EC-shCtrl treated with PBS is expected to display the strongest signal for total and phospho-protein levels, as it represents the baseline, intact activation state of the ANG2–TIE2 signaling pathway in control endothelial cells. The reduced signals observed in the other groups reflect genetic or pharmacologic perturbations of this pathway rather than nonspecific activation in the control condition. Regarding the concern about the correspondence between Figures 5E and 5F, the quantification presented in Figure 5F (panel C) was directly derived from the blots shown in Figure 5E. Densitometric analysis was initially performed using FUSION FX software and independently repeated using ImageJ, yielding the same overall trend as presented in Figure 5F(C). These analyses confirm that the quantified data accurately reflect the band intensities shown in the representative blots.

2. MAPK ERK pathway is known to act upstream by VEGF signaling to induce angiopoietin-2 expression. Additional experiments are needed to substantiate ERK activation downstream of EV activation and more importantly angiopoietin-2.

We thank the reviewer for this important comment. We agree that the ANG–TIE signaling network is complex and is regulated by multiple upstream mediators, including VEGF, with the MAPK/ERK pathway known to participate in ANG2 regulation in certain contexts. In the current study, our data support activation of ERK downstream of EV-mediated ANG2–TIE2 signaling; however, we acknowledge that the broader interplay between VEGF signaling, ERK activation, and ANG2 regulation was not fully dissected. Elucidating the hierarchical relationship between these pathways would require additional targeted experiments and is beyond the scope of the present study. We have now clarified this limitation and expanded the Discussion to better contextualize our findings within the broader VEGF–ERK–ANG2 signaling framework. (see discussion section, page 16, rows 383-401).

3. The Western blots lacked the total protein levels of AKT and ERK. The phosphorylation levels cannot be interpreted. Please show the total protein levels of the AKT and ERK.

We thank the reviewer for his comment. We added the total proteins levels of AKT and ERK to the western blots in Figure 5. (see new figure 5)

4. To substantiate the sufficiency of aniogpoietin-2 in angiogenesis, angiopoietin-2 alone needs to be added to the endothelial cells and monitored for proliferation, migration, invasion, tube formation, and pathway activation.

We thank the reviewer for his comment. We performed a new set of experiments using recombinant hANG2 and endothelial cells showing the effect of ANG2 alone on migration, invasion and tube formation. The data was added to the text and now depicted in new figure 3:

"To further support the sufficiency of ANG2 in promoting angiogenesis, we repeated the functional assays using recombinant human ANG2 (huANG2). Treatment with huANG2 significantly enhanced endothelial cell migration and invasion by 2.14-fold (p = 0.007) and 1.8-fold (p = 0.01), respectively (Figures 3E, 3F). In addition, huANG2 treatment increased endothelial tube formation by 2.83-fold compared with controls (p = 0.004; Figure 3G). Collectively, these results support a direct pro-angiogenic role for ANG2 and demonstrate that Met-EVs stimulate multiple pro-angiogenic behaviors in endothelial cells." (see results section, page 10, rows 241-247 and new figure 3).

5. The level of angiopoietin can be critical for its role between cell death and angiogenesis. Is the treatment of EVs Angiopoietin-2 at physiological doses?

We thank the reviewer for this important comment. Indeed, the level of angiopoietin can be critical in determining its biological effects, including the balance between pro-angiogenic signaling and other cellular outcomes. Based on the literature, recombinant ANG2 is commonly used in endothelial angiogenesis assays at concentrations ranging from 100–400 ng/mL in migration, invasion, and tube formation experiments, while higher concentrations (~800 ng/mL) have been reported to strongly activate TIE2 and downstream PI3K/Akt signaling [1-3]. In our experiments, we used 200 ng/mL recombinant ANG2, which falls within the commonly used physiological range reported in the literature for in vitro endothelial angiogenesis assays. This information has now been clarified in the revised manuscript.

Minor Concerns:

7. Perform the protein analysis in Figure 4 for the shANG2-metEVs to understand if other factors not just angiopoietin-2 are being reduced.

We thank the reviewer for this thoughtful suggestion. In Figure 4, we performed a comprehensive proteomic angiogenesis profiling of WT Met-EVs to identify angiogenesis-related proteins present in these vesicles, which revealed ANG2 as one of the most abundant pro-angiogenic factors. To specifically evaluate the role of ANG2, we subsequently generated shANG2 Met-EVs and confirmed the efficient reduction of ANG2 in these vesicles by Western blot analysis. Our intention in this part of the study was to selectively reduce ANG2 levels in Met-EVs to functionally assess its contribution to the pro-angiogenic effects observed. Because the knockdown strategy specifically targets ANG2 expression, and we confirmed its reduction at the protein level in EVs, repeating the full angiogenesis proteomic array for shANG2 Met-EVs would not provide additional mechanistic insight into the specific role of ANG2 that we aimed to address. Therefore, we focused on validating ANG2 depletion and examining the resulting functional consequences in endothelial cells.

We hope this clarification explains our experimental design and rationale.

8. Abstract and Introduction (Line 60) both begin discussing GI cancers. The introduction also describes ovarian cancer. This clouds the focus of the paper on mesothelial cells and their role in angiogenesis. Begin discussing the importance of mesothelial cells and their role in angiogenesis for GI and ovarian cancers.

We thank the reviewer for this helpful comment. In response, we revised the Abstract and Introduction to place greater emphasis on the role of peritoneal mesothelial cells in regulating angiogenesis within the peritoneal tumor microenvironment of gastrointestinal and ovarian cancers. These changes clarify the central focus of the study and improve the overall narrative of the manuscript.

The revised text was added to the abstract:

"Peritoneal mesothelial cells play a critical role in shaping the peritoneal tumor microenvironment and are increasingly recognized as active regulators of angiogenesis in cancers that metastasize to the peritoneum, including gastrointestinal (GI) and ovarian malignancies. Through complex interactions with tumor and stromal cells, PMCs contribute to the establishment of a pro-metastatic niche in the peritoneal cavity. Extracellular vesicles (EVs), nanosized vesicles secreted by most cell types, mediate intercellular communication by transferring bioactive molecules such as proteins, lipids, and nucleic acids. While tumor-derived EVs have been extensively studied in cancer progression, the role of PMC-derived EVs in regulating endothelial function and angiogenesis remains largely unexplored." (see abstract, page 2, rows 31-40).

The revised text was added to the introduction:

"Peritoneal mesothelial cells form the cellular lining of the peritoneal cavity and play a central role in regulating the peritoneal tumor microenvironment. Increasing evidence suggests that peritoneal mesothelial cells actively contribute to the establishment of a pro-metastatic niche in cancers that disseminate within the peritoneal cavity, including gastrointestinal and ovarian malignancies. The peritoneal cavity is a frequent site of dissemination for these cancers, and current systemic therapies remain largely ineffective in controlling peritoneal metastasis originating from the pancreas, stomach, colon, or ovary. Consequently, once peritoneal spread occurs, it is associated with aggressive tumor progression, rapid clinical decline, and poor survival [1–4]. Following detachment from the primary tumor, cancer cells can enter the peritoneal cavity through peritoneal fluid and adhere to the mesothelial surface, where they interact with peritoneal mesothelial cells and establish secondary lesions [5,6]. Beneath the mesothelial layer lies the peritoneal membrane, which contains fibroblasts, immune cells such as macrophages and mast cells, as well as vascular and lymphatic networks. After attaching to mesothelial cells, tumor cells can penetrate this barrier and invade underlying tissues, including the omentum and bowel serosa. Angiogenesis, the formation of new blood vessels from pre-existing vasculature, is a critical process supporting the growth and survival of both primary and metastatic tumors. During angiogenesis, endothelial cells (ECs) proliferate, migrate, and organize into tubular structures, ultimately enabling neovascularization [7]. In intraperitoneal tumors, which disseminate primarily by direct seeding rather than hematogenous spread, enhanced vascularization of metastatic sites is a key determinant of tumor progression and survival." (see introduction, page 3, rows 60-80).

9. Line 78 and 79: Introduction states that angiopoietin-2 lies upstream and activates of PI3K, AKT, and ERK. The author cites 3 papers. These papers show the sufficiency of angiopoietin-2 to activate PI3K and AKT at high concentrations. There is no documentation of ERK phosphorylation. Most of the published literature supports ERK activation as upstream of angiopoietin-2 transcription through VEGF signaling.

We thank the reviewer for this important comment. Previous studies have demonstrated that ANG2 signaling through the TIE2 receptor can activate multiple downstream pathways in endothelial cells, including the PI3K/AKT and MAPK/ERK signaling cascades, which regulate endothelial cell survival, migration, and angiogenic responses. ERK phosphorylation following ANG2 stimulation has been reported in endothelial cells and is considered part of the downstream signaling network associated with ANG–TIE2 activation [4, 5]. The relevant references have now been added to the manuscript (see References 13 and 14).

10. 5Fa and 5Fc label relative expression on the y-axis. It should be listed as relative signal as pERK and pAKT is examining for phosphorylation and not protein levels.

We thank the reviewer for this helpful comment. In response, the y-axis labels in Figures 5F(a) and 5F(c) have been revised from “relative expression” to “relative signal” to more accurately reflect that pERK and pAKT represent phosphorylation levels rather than total protein expression.

11. Line 280-281: An alternative explanation is that the inhibition of TIE-2 blocked the function of Angiopoietin-1.

We thank the reviewer for this insightful comment. We acknowledge that TIE2 inhibition could also affect ANG1-mediated signaling. We have now clarified this point in the revised manuscript to acknowledge this potential alternative explanation.

The text was added to the discussion:

" Functional tube-formation assays further demonstrated that ANG2-containing EVs restore angiogenic capacity in ANG2-deficient ECs, an effect that was abolished following pharmacological inhibition of TIE2. Although TIE2 inhibition may also interfere with signaling mediated by other ligands such as ANG1, these results nonetheless support a central role for the ANG2–TIE2 signaling axis in mediating the pro-angiogenic effects of mesothelial EVs in ECs." (see discussion, page 15, rows 368-373).

12. Angiopoietin-2 alone promotes cell death but with VEGF promotes angiogenesis. However, Figure 4 shows low level of VEGF in comparison. How do explain this angiogenic role of angiopoietin-2 in this system?

We thank the reviewer for this insightful comment. Indeed, the biological activity of ANG2 is context-dependent, and previous studies have shown that ANG2 can promote endothelial cell destabilization and apoptosis in the absence of pro-angiogenic stimuli such as VEGF, while in the presence of VEGF it facilitates angiogenic remodeling. However, angiogenesis is regulated by a complex network of signaling pathways, and ANG2 activity can also be supported by additional pro-angiogenic factors and signaling inputs. In our proteomic profiling (Figure 4), although VEGF levels were relatively low, several other pro-angiogenic mediators were detected in Met-EVs, including ANG1, IL-8, GM-CSF, and uPAR, all of which have been reported to promote endothelial activation and angiogenesis. These factors may cooperate with ANG2 to support endothelial survival and angiogenic behavior in our system. Moreover, ANG2 has been reported to activate endothelial signaling through both the TIE2 receptor and integrin pathways, which can stimulate downstream signaling such as PI3K/AKT and MAPK/ERK independent of VEGF. Therefore, while VEGF is a well-known partner of ANG2 in angiogenesis, the angiogenic phenotype observed in our study is likely driven by the combined activity of multiple pro-angiogenic mediators present in Met-EVs together with ANG2 signaling. We have now clarified this point in the revised discussion (see new revised discussion, page 16, rows 396-401).

13. Discussion lacks an in-depth presentation of the interplay between angiopoietin 1 and 2 and other angiogenic factors like VEGF.

We thank the reviewer for this valuable comment. In response, we have expanded and revised the Discussion to provide a more in-depth description of the interplay between ANG1, ANG2, and other angiogenic mediators, including VEGF, within the ANG–TIE signaling network. These revisions also incorporate related points raised by this reviewer and the other reviewers to better contextualize our findings within the broader angiogenic signaling landscape (see new revised discussion, page 16, rows 383-401).

1.	Akwii, R.G., et al., *Role of Angiopoietin-2 in Vascular Physiology and Pathophysiology.* Cells, 2019. **8**(5).

2.	Kim, I., et al., *Angiopoietin-2 at high concentration can enhance endothelial cell survival through the phosphatidylinositol 3'-kinase/Akt signal transduction pathway.* Oncogene, 2000. **19**(39): p. 4549-52.

3.	Sim, M., et al., *Angiopoietin-2 binds to FGFR2, inhibits FGF-FGFR2 signaling, and delays cutaneous wound healing by inhibiting wound angiogenesis.* Angiogenesis, 2025. **28**(4): p. 43.

4.	Harfouche, R. and S.N. Hussain, *Signaling and regulation of endothelial cell survival by angiopoietin-2.* Am J Physiol Heart Circ Physiol, 2006. **291**(4): p. H1635-45.

5.	Li, S., et al., *Differential roles of p38 MAPK and ERK1/2 in angiopoietin-2-mediated rat pulmonary microvascular endothelial cell apoptosis induced by lipopolysaccharide.* Exp Ther Med, 2018. **16**(6): p. 4729-4736.
