# Supplementary material for: Mesothelial cells derived extracellular vesicles promote angiogenesis through the transfer of angiopoietin-2
Source: PLoS One. 2026 Jul 15;21(7):e0353115. doi: 10.1371/journal.pone.0353115 (PMC13372138; doi:10.1371/journal.pone.0353115)
Supplement: Peer review history

# Author comment: Author response to Decision Letter 0

**DOI:** 10.1371/journal.pone.0353115.r001

10 Sep 2025

Attachment Submitted filename: Response letter PlosONE 3.9.25.docx

# Peer review: Decision Letter 0

**DOI:** 10.1371/journal.pone.0353115.r002

**Authors:** Yu An

5 Nov 2025

PONE-D-25-48703

Mesothelial cells derived extracellular vesicles promote angiogenesis through the transfer of angiopoietin-2

PLOS ONE

Dear Dr. Loewenstein,

Thank you for submitting your manuscript to PLOS ONE. After careful consideration, we feel that it has merit but does not fully meet PLOS ONE’s publication criteria as it currently stands. Therefore, we invite you to submit a revised version of the manuscript that addresses the points raised during the review process.

Two invited reviewers have raised several shared concerns about the current manuscript, and therefore, a majorly revised version is warranted for a further consideration of this work.

Please submit your revised manuscript by Dec 20 2025 11:59PM. If you will need more time than this to complete your revisions, please reply to this message or contact the journal office at plosone@plos.org. When you're ready to submit your revision, log on to https://www.editorialmanager.com/pone/ and select the 'Submissions Needing Revision' folder to locate your manuscript file.

Please include the following items when submitting your revised manuscript:

- A rebuttal letter that responds to each point raised by the academic editor and reviewer(s). You should upload this letter as a separate file labeled 'Response to Reviewers'.
- A marked-up copy of your manuscript that highlights changes made to the original version. You should upload this as a separate file labeled 'Revised Manuscript with Track Changes'.
- An unmarked version of your revised paper without tracked changes. You should upload this as a separate file labeled 'Manuscript'.

If you would like to make changes to your financial disclosure, please include your updated statement in your cover letter. Guidelines for resubmitting your figure files are available below the reviewer comments at the end of this letter.

If applicable, we recommend that you deposit your laboratory protocols in protocols.io to enhance the reproducibility of your results. Protocols.io assigns your protocol its own identifier (DOI) so that it can be cited independently in the future. For instructions see: https://journals.plos.org/plosone/s/submission-guidelines#loc-laboratory-protocols. Additionally, PLOS ONE offers an option for publishing peer-reviewed Lab Protocol articles, which describe protocols hosted on protocols.io. Read more information on sharing protocols at https://plos.org/protocols?utm_medium=editorial-email&utm_source=authorletters&utm_campaign=protocols.

We look forward to receiving your revised manuscript.

Kind regards,

Yu A. An, M.D., Ph.D.

Academic Editor

PLOS ONE

Journal Requirements:

When submitting your revision, we need you to address these additional requirements.

1. Please ensure that your manuscript meets PLOS ONE's style requirements, including those for file naming. The PLOS ONE style templates can be found at

https://journals.plos.org/plosone/s/file?id=wjVg/PLOSOne_formatting_sample_main_body.pdf and

https://journals.plos.org/plosone/s/file?id=ba62/PLOSOne_formatting_sample_title_authors_affiliations.pdf.

2. To comply with PLOS One submissions requirements, in your Methods section, please provide additional information regarding the experiments involving animals and ensure you have included details on (1) methods of sacrifice, (2) methods of anesthesia and/or analgesia, and (3) efforts to alleviate suffering.

3. We noticed you have some minor occurrence of overlapping text with the following previous publication(s), which needs to be addressed:

https://doi.org/10.3390/cancers14122953

In your revision ensure you cite all your sources (including your own works), and quote or rephrase any duplicated text outside the methods section. Further consideration is dependent on these concerns being addressed.

4. We note that your Data Availability Statement is currently as follows: [All relevant data are within the manuscript and its Supporting Information files.]

Please confirm at this time whether or not your submission contains all raw data required to replicate the results of your study. Authors must share the “minimal data set” for their submission. PLOS defines the minimal data set to consist of the data required to replicate all study findings reported in the article, as well as related metadata and methods (https://journals.plos.org/plosone/s/data-availability#loc-minimal-data-set-definition).

For example, authors should submit the following data:

- The values behind the means, standard deviations and other measures reported;

- The values used to build graphs;

- The points extracted from images for analysis.

Authors do not need to submit their entire data set if only a portion of the data was used in the reported study.

If your submission does not contain these data, please either upload them as Supporting Information files or deposit them to a stable, public repository and provide us with the relevant URLs, DOIs, or accession numbers. For a list of recommended repositories, please see https://journals.plos.org/plosone/s/recommended-repositories.

If there are ethical or legal restrictions on sharing a de-identified data set, please explain them in detail (e.g., data contain potentially sensitive information, data are owned by a third-party organization, etc.) and who has imposed them (e.g., an ethics committee). Please also provide contact information for a data access committee, ethics committee, or other institutional body to which data requests may be sent. If data are owned by a third party, please indicate how others may request data access.

5. PLOS ONE now requires that authors provide the original uncropped and unadjusted images underlying all blot or gel results reported in a submission’s figures or Supporting Information files. This policy and the journal’s other requirements for blot/gel reporting and figure preparation are described in detail at https://journals.plos.org/plosone/s/figures#loc-blot-and-gel-reporting-requirements and https://journals.plos.org/plosone/s/figures#loc-preparing-figures-from-image-files. When you submit your revised manuscript, please ensure that your figures adhere fully to these guidelines and provide the original underlying images for all blot or gel data reported in your submission. See the following link for instructions on providing the original image data: https://journals.plos.org/plosone/s/figures#loc-original-images-for-blots-and-gels.

In your cover letter, please note whether your blot/gel image data are in Supporting Information or posted at a public data repository, provide the repository URL if relevant, and provide specific details as to which raw blot/gel images, if any, are not available. Email us at plosone@plos.org if you have any questions.

6. If the reviewer comments include a recommendation to cite specific previously published works, please review and evaluate these publications to determine whether they are relevant and should be cited. There is no requirement to cite these works unless the editor has indicated otherwise.

[Note: HTML markup is below. Please do not edit.]

Reviewers' comments:

Reviewer's Responses to Questions

**Comments to the Author**

1. Is the manuscript technically sound, and do the data support the conclusions?

The manuscript must describe a technically sound piece of scientific research with data that supports the conclusions. Experiments must have been conducted rigorously, with appropriate controls, replication, and sample sizes. The conclusions must be drawn appropriately based on the data presented.

Reviewer #1: Yes

Reviewer #2: No

**********

2. Has the statistical analysis been performed appropriately and rigorously?

Reviewer #1: Yes

Reviewer #2: I Don't Know

**********

3. Have the authors made all data underlying the findings in their manuscript fully available?

The PLOS Data policy requires authors to make all data underlying the findings described in their manuscript fully available without restriction, with rare exception (please refer to the Data Availability Statement in the manuscript PDF file). The data should be provided as part of the manuscript or its supporting information, or deposited to a public repository. For example, in addition to summary statistics, the data points behind means, medians and variance measures should be available. If there are restrictions on publicly sharing data—e.g. participant privacy or use of data from a third party—those must be specified.

Reviewer #1: No

Reviewer #2: Yes

**********

4. Is the manuscript presented in an intelligible fashion and written in standard English?

PLOS ONE does not copyedit accepted manuscripts, so the language in submitted articles must be clear, correct, and unambiguous. Any typographical or grammatical errors should be corrected at revision, so please note any specific errors here.

Reviewer #1: Yes

Reviewer #2: Yes

**********

5. Review Comments to the Author

Please use the space provided to explain your answers to the questions above. You may also include additional comments for the author, including concerns about dual publication, research ethics, or publication ethics. (Please upload your review as an attachment if it exceeds 20,000 characters)

Reviewer #1: The manuscript provides strong evidence that mesothelial cell–derived extracellular vesicles (EVs) enhance angiogenesis via ANG2–TIE2 signaling. The study is well-designed, methodologically sound, and relevant to peritoneal metastasis research. Some clarifications and refinements are required before publication.

Major Revisions:

• Clarify Novelty

Clearly describe how this work differs from Kalfon et al., 2022, specifying new experiments (e.g., in vivo Matrigel plug assay, ANG2 knockdown) and novel mechanistic findings.

• Mechanistic Evidence

Note that direct receptor-binding validation (e.g., co-IP or blocking assay) was not performed; acknowledge this as a limitation and suggest future confirmation.

• Data and Figure Transparency

Improve the resolution and labeling of Figures 3 and 5. Include biological replicates (n), statistical tests used, and exact p-values in figure legends. Specify whether error bars represent SD or SEM.

• Animal Study Details

Provide group sample sizes, information on randomization/blinding, and add a statement confirming compliance with ARRIVE guidelines.

Minor Revisions:

• Abstract

Mention the identification of 43 angiogenic regulators and emphasize ANG2 as the key mediator. Conclude with a sentence on the therapeutic potential of targeting ANG2.

• Terminology and Formatting

Ensure consistent use of abbreviations (EVs, Met-EVs, PMCs, ECs) and standardized symbols (µg, µM). Maintain uniform formatting of gene and protein names.

• References

Verify formatting and remove duplicates (especially #15 and #26). Add at least one recent (2023–2024) reference on EV-mediated angiogenesis.

• Language and Style

Shorten long sentences in the Discussion (lines 296–350) for clarity. Maintain a consistent academic tone and correct minor typographical errors.

Overall Evaluation:

A well-executed and valuable study that advances understanding of mesothelial–endothelial communication in peritoneal metastasis. Addressing these points will enhance clarity, reproducibility, and overall impact. The manuscript will be suitable for publication following minor revision.

Reviewer #2: The authors present evidence that angiopoietin-2 is present in met-EVs and these EVs are taken up by endothelial cells. The experiments show a necessity of angiopoietin-2 through in vitro assays for cell proliferation, migration, invasion, and tube formation. However, the current manuscript lacks the necessary experiments to mechanistically and physiologically discern the role of angiopoietin-2 in cell signaling and to control for its interplay with angiopoietin-1, VEGF, and milieu of other met-EV factors.

Major Concerns:

1. EC-shCtrl treated with PBS show the strongest signal for all protein and phospho-protein levels. Such a robust signal suggests a nonspecific or artificial activation of the pathway. Furthermore, the signals in 5F do not visually match the 5E blots.

2. MAPK ERK pathway is known to act upstream by VEGF signaling to induce angiopoietin-2 expression. Additional experiments are needed to substantiate ERK activation downstream of EV activation and more importantly angiopoietin-2. Such as, perform both dose dependent and time dependent treatments of EVs and angiopoietin-2 alone to HUVECs with the necessary positive controls.

3. The Western blots lacked the total protein levels of AKT and ERK. The phosphorylation levels cannot be interpreted. Please show the total protein levels of the AKT and ERK.

4. To substantiate the sufficiency of aniogpoietin-2 in angiogenesis, angiopoietin-2 alone needs to be added to the endothelial cells and monitored for proliferation, migration, invasion, tube formation, and pathway activation.

5. The level of angiopoietin can be critical for its role between cell death and angiogenesis. Is the treatment of EVs Angiopoietin-2 at physiological doses?

6. If not, are these angiogenic results driven by an artificial environment by over expression or over dosing of angiopoietin-2?

Minor Concerns:

7. Perform the protein analysis in Figure 4 for the shANG2-metEVs to understand if other factors not just angiopoietin-2 are being reduced.

8. Abstract and Introduction (Line 60) both begin discussing GI cancers. The introduction also describes ovarian cancer. This clouds the focus of the paper on mesothelial cells and their role in angiogenesis. Begin discussing the importance of mesothelial cells and their role in angiogenesis for GI and ovarian cancers.

9. Line 78 and 79: Introduction states that angiopoietin-2 lies upstream and activates of PI3K, AKT, and ERK. The author cites 3 papers. These papers show the sufficiency of angiopoietin-2 to activate PI3K and AKT at high concentrations. There is no documentation of ERK phosphorylation. The majority of the published literature supports ERK activation as upstream of angiopoietin-2 transcription through VEGF signaling.

10. 5Fa and 5Fc label relative expression on the y-axis. It should be listed as relative signal as pERK and pAKT is examining for phosphorylation and not protein levels.

11. Line 280-281: An alternative explanation is that the inhibition of TIE-2 blocked the function of Angiopoietin-1.

12. Angiopoietin-2 alone promotes cell death but with VEGF promotes angiogenesis. However, Figure 4 shows low level of VEGF in comparison. How do explain this angiogenic role of angiopoietin-2 in this system?

13. Discussion lacks an indepth presentation of the interplay between angiopoietin 1 and 2 and other angiogenic factors like VEGF.

**********

6. PLOS authors have the option to publish the peer review history of their article (what does this mean?). If published, this will include your full peer review and any attached files.

If you choose “no”, your identity will remain anonymous but your review may still be made public.

**Do you want your identity to be public for this peer review?**   For information about this choice, including consent withdrawal, please see our Privacy Policy.

Reviewer #1: **Yes:**  DIBYASHREE CHHETRI

Reviewer #2: No

**********

[NOTE: If reviewer comments were submitted as an attachment file, they will be attached to this email and accessible via the submission site. Please log into your account, locate the manuscript record, and check for the action link "View Attachments". If this link does not appear, there are no attachment files.]

To ensure your figures meet our technical requirements, please review our figure guidelines: https://journals.plos.org/plosone/s/figures

You may also use PLOS’s free figure tool, NAAS, to help you prepare publication quality figures: https://journals.plos.org/plosone/s/figures#loc-tools-for-figure-preparation.

NAAS will assess whether your figures meet our technical requirements by comparing each figure against our figure specifications.

# Author comment: Author response to Decision Letter 1

**DOI:** 10.1371/journal.pone.0353115.r003

27 Apr 2026

Reviewer #1: The manuscript provides strong evidence that mesothelial cell–derived extracellular vesicles (EVs) enhance angiogenesis via ANG2–TIE2 signaling. The study is well-designed, methodologically sound, and relevant to peritoneal metastasis research. Some clarifications and refinements are required before publication.

Major Revisions:

1. Clarify Novelty

Clearly describe how this work differs from Kalfon et al., 2022, specifying new experiments (e.g., in vivo Matrigel plug assay, ANG2 knockdown) and novel mechanistic findings.

We thank the reviewer for this important comment. Kalfon et al. (2022) focused on the pro-angiogenic effects of extracellular vesicles (EVs) derived from tumor cells (i.e., gastric cancer cells). In contrast, the present study investigates the angiogenic effects of EVs derived from cells within the tumor microenvironment and the metastatic peritoneal niche, specifically mesothelial cells. Thus, while the previous work addressed tumor cell–derived EVs, the current study shifts the focus to stromal components and their contribution to angiogenesis. In both studies, ANG2 was identified as one of the most abundant pro-angiogenic proteins present in EVs. However, the biological context and mechanistic implications differ significantly. In the current work, we demonstrate for the first time that mesothelial cell–derived EVs actively promote angiogenesis, highlighting their role in shaping the pre-metastatic niche and facilitating metastatic progression. Moreover, this study extends beyond our prior work by incorporating additional experimental approaches, including the in vivo Matrigel plug assay using the ANG2 knockdown EVs, which provide mechanistic evidence supporting the functional role of mesothelial EV-derived ANG2 in angiogenesis. Together, these findings establish a novel role for mesothelial cell–derived EVs in promoting angiogenesis as part of the contribution of the pre-metastatic niche to metastatic progression, thereby clearly distinguishing this work from Kalfon et al. (2022).

The comment was addressed, and a short paragraph was added to the discussion section (see discussion section, page 15, row 353-362).

2. Mechanistic Evidence

Note that direct receptor-binding validation (e.g., co-IP or blocking assay) was not performed; acknowledge this as a limitation and suggest future confirmation.

Although we did not perform direct receptor-binding validation such as co-immunoprecipitation (co-IP), we did conduct functional blocking experiments using a TIE2 inhibitor (TIE2-I). As shown in Figures 5E and 5F, endothelial cells lacking ANG2 and treated with ANG2-deficient EVs exhibited reduced activation of the PI3K/Akt and ERK signaling pathways. Rescue with ANG2-positive EVs restored pathway activity, whereas inhibition of TIE2 abrogated this effect. (see results section, page 11, rows 274-277 and figure 5E and 5F).

While these findings functionally support the involvement of the ANG2–TIE2 axis, we acknowledge that direct receptor-binding assays were not performed and represent a limitation of the current study. Future studies incorporating co-IP or additional receptor-binding approaches will be important to further validate this interaction at the molecular level.

The comment was addressed, and a short paragraph was added to the discussion section (see discussion section, page 15, row 373-376).

3. Data and Figure Transparency

Improve the resolution and labeling of Figures 3 and 5. Include biological replicates (n), statistical tests used, and exact p-values in figure legends. Specify whether error bars represent SD or SEM.

We thank the reviewer for this helpful comment. Figures 3 and 5 have been revised to improve resolution and labeling. In addition, where information was previously missing, we have updated the figure legends to include the number of biological replicates (n), the statistical tests used, the exact p-values, and a clear specification of whether error bars represent SD or SEM. Revised, higher-resolution versions of Figures 3 and 5 have been incorporated into the manuscript.

4. Animal Study Details

Provide group sample sizes, information on randomization/blinding, and add a statement confirming compliance with ARRIVE guidelines.

We thank the reviewer for this important comment. Group sample sizes are now clearly indicated in the legend of Figure 6 and have also been added to the Matrigel Plug Assay in Mice subsection in the Materials and Methods section. We further clarify that all mice were randomly assigned to experimental groups. Whenever feasible, investigators were blinded to group allocation during data collection and analysis. In addition, we have included a statement confirming that all animal experiments were conducted in accordance with ARRIVE guidelines. (see materials and methods section, page 7, row 174-179 and figure 6 legend).

Minor Revisions:

1.Abstract

Mention the identification of 43 angiogenic regulators and emphasize ANG2 as the key mediator. Conclude with a sentence on the therapeutic potential of targeting ANG2.

We thank the reviewer for this thoughtful suggestion. These points are now clearly articulated in the revised abstract to better reflect the scope of our findings and the potential clinical relevance of targeting ANG2.

2. Terminology and Formatting

Ensure consistent use of abbreviations (EVs, Met-EVs, PMCs, ECs) and standardized symbols (µg, µM). Maintain uniform formatting of gene and protein names.

We thank the reviewer for this careful observation. The manuscript has been thoroughly reviewed to ensure consistent use of abbreviations (EVs, Met-EVs, PMCs, ECs) and standardized units (µg, µM) throughout. In addition, gene and protein names have been uniformly formatted according to standard conventions. All necessary corrections have been implemented in the revised version of the manuscript.

3. References

Verify formatting and remove duplicates (especially #15 and #26). Add at least one recent (2023–2024) reference on EV-mediated angiogenesis.

We thank the reviewer for this helpful comment. The reference list has been carefully reviewed to verify formatting consistency. In addition, we have incorporated three recent references (2024–2025) addressing EV-mediated angiogenesis to ensure the manuscript reflects the most up-to-date literature in the field (references 20-22).

4. Language and Style

Shorten long sentences in the Discussion (lines 296–350) for clarity. Maintain a consistent academic tone and correct minor typographical errors.

We thank the reviewer for this helpful comment. The Discussion section has been carefully revised to shorten long sentences and improve overall clarity and readability. We have also ensured a consistent academic tone throughout and corrected minor typographical errors in the revised manuscript.

Reviewer #2: The authors present evidence that angiopoietin-2 is present in met-EVs and these EVs are taken up by endothelial cells. The experiments show a necessity of angiopoietin-2 through in vitro assays for cell proliferation, migration, invasion, and tube formation. However, the current manuscript lacks the necessary experiments to mechanistically and physiologically discern the role of angiopoietin-2 in cell signaling and to control for its interplay with angiopoietin-1, VEGF, and milieu of other met-EV factors.

Major Concerns:

1. EC-shCtrl treated with PBS show the strongest signal for all protein and phospho-protein levels. Such a robust signal suggests a nonspecific or artificial activation of the pathway. Furthermore, the signals in 5F do not visually match the 5E blots.

We thank the reviewer for this important comment. EC-shCtrl treated with PBS is expected to display the strongest signal for total and phospho-protein levels, as it represents the baseline, intact activation state of the ANG2–TIE2 signaling pathway in control endothelial cells. The reduced signals observed in the other groups reflect genetic or pharmacologic perturbations of this pathway rather than nonspecific activation in the control condition. Regarding the concern about the correspondence between Figures 5E and 5F, the quantification presented in Figure 5F (panel C) was directly derived from the blots shown in Figure 5E. Densitometric analysis was initially performed using FUSION FX software and independently repeated using ImageJ, yielding the same overall trend as presented in Figure 5F(C). These analyses confirm that the quantified data accurately reflect the band intensities shown in the representative blots.

2. MAPK ERK pathway is known to act upstream by VEGF signaling to induce angiopoietin-2 expression. Additional experiments are needed to substantiate ERK activation downstream of EV activation and more importantly angiopoietin-2.

We thank the reviewer for this important comment. We agree that the ANG–TIE signaling network is complex and is regulated by multiple upstream mediators, including VEGF, with the MAPK/ERK pathway known to participate in ANG2 regulation in certain contexts. In the current study, our data support activation of ERK downstream of EV-mediated ANG2–TIE2 signaling; however, we acknowledge that the broader interplay between VEGF signaling, ERK activation, and ANG2 regulation was not fully dissected. Elucidating the hierarchical relationship between these pathways would require additional targeted experiments and is beyond the scope of the present study. We have now clarified this limitation and expanded the Discussion to better contextualize our findings within the broader VEGF–ERK–ANG2 signaling framework. (see discussion section, page 16, rows 383-401).

3. The Western blots lacked the total protein levels of AKT and ERK. The phosphorylation levels cannot be interpreted. Please show the total protein levels of the AKT and ERK.

We thank the reviewer for his comment. We added the total proteins levels of AKT and ERK to the western blots in Figure 5. (see new figure 5)

4. To substantiate the sufficiency of aniogpoietin-2 in angiogenesis, angiopoietin-2 alone needs to be added to the endothelial cells and monitored for proliferation, migration, invasion, tube formation, and pathway activation.

We thank the reviewer for his comment. We performed a new set of experiments using recombinant hANG2 and endothelial cells showing the effect of ANG2 alone on migration, invasion and tube formation. The data was added to the text and now depicted in new figure 3:

"To further support the sufficiency of ANG2 in promoting angiogenesis, we repeated the functional assays using recombinant human ANG2 (huANG2). Treatment with huANG2 significantly enhanced endothelial cell migration and invasion by 2.14-fold (p = 0.007) and 1.8-fold (p = 0.01), respectively (Figures 3E, 3F). In addition, huANG2 treatment increased endothelial tube formation by 2.83-fold compared with controls (p = 0.004; Figure 3G). Collectively, these results support a direct pro-angiogenic role for ANG2 and demonstrate that Met-EVs stimulate multiple pro-angiogenic behaviors in endothelial cells." (see results section, page 10, rows 241-247 and new figure 3).

5. The level of angiopoietin can be critical for its role between cell death and angiogenesis. Is the treatment of EVs Angiopoietin-2 at physiological doses?

We thank the reviewer for this important comment. Indeed, the level of angiopoietin can be critical in determining its biological effects, including the balance between pro-angiogenic signaling and other cellular outcomes. Based on the literature, recombinant ANG2 is commonly used in endothelial angiogenesis assays at concentrations ranging from 100–400 ng/mL in migration, invasion, and tube formation experiments, while higher concentrations (~800 ng/mL) have been reported to strongly activate TIE2 and downstream PI3K/Akt signaling [1-3]. In our experiments, we used 200 ng/mL recombinant ANG2, which falls within the commonly used physiological range reported in the literature for in vitro endothelial angiogenesis assays. This information has now been clarified in the revised manuscript.

Minor Concerns:

7. Perform the protein analysis in Figure 4 for the shANG2-metEVs to understand if other factors not just angiopoietin-2 are being reduced.

We thank the reviewer for this thoughtful suggestion. In Figure 4, we performed a comprehensive proteomic angiogenesis profiling of WT Met-EVs to identify angiogenesis-related proteins present in these vesicles, which revealed ANG2 as one of the most abundant pro-angiogenic factors. To specifically evaluate the role of ANG2, we subsequently generated shANG2 Met-EVs and confirmed the efficient reduction of ANG2 in these vesicles by Western blot analysis. Our intention in this part of the study was to selectively reduce ANG2 levels in Met-EVs to functionally assess its contribution to the pro-angiogenic effects observed. Because the knockdown strategy specifically targets ANG2 expression, and we confirmed its reduction at the protein level in EVs, repeating the full angiogenesis proteomic array for shANG2 Met-EVs would not provide additional mechanistic insight into the specific role of ANG2 that we aimed to address. Therefore, we focused on validating ANG2 depletion and examining the resulting functional consequences in endothelial cells.

We hope this clarification explains our experimental design and rationale.

8. Abstract and Introduction (Line 60) both begin discussing GI cancers. The introduction also describes ovarian cancer. This clouds the focus of the paper on mesothelial cells and their role in angiogenesis. Begin discussing the importance of mesothelial cells and their role in angiogenesis for GI and ovarian cancers.

We thank the reviewer for this helpful comment. In response, we revised the Abstract and Introduction to place greater emphasis on the role of peritoneal mesothelial cells in regulating angiogenesis within the peritoneal tumor microenvironment of gastrointestinal and ovarian cancers. These changes clarify the central focus of the study and improve the overall narrative of the manuscript.

The revised text was added to the abstract:

"Peritoneal mesothelial cells play a critical role in shaping the peritoneal tumor microenvironment and are increasingly recognized as active regulators of angiogenesis in cancers that metastasize to the peritoneum, including gastrointestinal (GI) and ovarian malignancies. Through complex interactions with tumor and stromal cells, PMCs contribute to the establishment of a pro-metastatic niche in the peritoneal cavity. Extracellular vesicles (EVs), nanosized vesicles secreted by most cell types, mediate intercellular communication by transferring bioactive molecules such as proteins, lipids, and nucleic acids. While tumor-derived EVs have been extensively studied in cancer progression, the role of PMC-derived EVs in regulating endothelial function and angiogenesis remains largely unexplored." (see abstract, page 2, rows 31-40).

The revised text was added to the introduction:

"Peritoneal mesothelial cells form the cellular lining of the peritoneal cavity and play a central role in regulating the peritoneal tumor microenvironment. Increasing evidence suggests that peritoneal mesothelial cells actively contribute to the establishment of a pro-metastatic niche in cancers that disseminate within the peritoneal cavity, including gastrointestinal and ovarian malignancies. The peritoneal cavity is a frequent site of dissemination for these cancers, and current systemic therapies remain largely ineffective in controlling peritoneal metastasis originating from the pancreas, stomach, colon, or ovary. Consequently, once peritoneal spread occurs, it is associated with aggressive tumor progression, rapid clinical decline, and poor survival [1–4]. Following detachment from the primary tumor, cancer cells can enter the peritoneal cavity through peritoneal fluid and adhere to the mesothelial surface, where they interact with peritoneal mesothelial cells and establish secondary lesions [5,6]. Beneath the mesothelial layer lies the peritoneal membrane, which contains fibroblasts, immune cells such as macrophages and mast cells, as well as vascular and lymphatic networks. After attaching to mesothelial cells, tumor cells can penetrate this barrier and invade

Attachment Submitted filename: Response letter-Plos ONE-15.3.26.docx

# Peer review: Decision Letter 1

**DOI:** 10.1371/journal.pone.0353115.r004

**Authors:** Yu An, Yu An

29 May 2026

PONE-D-25-48703R1

Mesothelial cells derived extracellular vesicles promote angiogenesis through the transfer of angiopoietin-2

PLOS One

Dear Dr. Loewenstein,

Thank you for submitting your manuscript to PLOS ONE. After careful consideration, we feel that it has merit but does not fully meet PLOS ONE’s publication criteria as it currently stands. Therefore, we invite you to submit a revised version of the manuscript that addresses the points raised during the review process.

In light of both reviewers' recommendations, your manuscript has been considered Accept in Principle, pending minor changes. Minor revisions including final language polishing, simplification of figure legends, and consistency in statistical reporting are recommended.

Please submit your revised manuscript by Jul 13 2026 11:59PM. If you will need more time than this to complete your revisions, please reply to this message or contact the journal office at plosone@plos.org. When you're ready to submit your revision, log on to https://www.editorialmanager.com/pone/ and select the 'Submissions Needing Revision' folder to locate your manuscript file.

Please include the following items when submitting your revised manuscript:

- A letter that responds to each point raised by the academic editor and reviewer(s). You should upload this letter as a separate file labeled 'Response to Reviewers'.
- A marked-up copy of your manuscript that highlights changes made to the original version. You should upload this as a separate file labeled 'Revised Manuscript with Track Changes'.
- An unmarked version of your revised paper without tracked changes. You should upload this as a separate file labeled 'Manuscript'.

If you would like to make changes to your financial disclosure, please include your updated statement in your cover letter. Guidelines for resubmitting your figure files are available below the reviewer comments at the end of this letter.

If applicable, we recommend that you deposit your laboratory protocols in protocols.io to enhance the reproducibility of your results. Protocols.io assigns your protocol its own identifier (DOI) so that it can be cited independently in the future. For instructions see: https://journals.plos.org/plosone/s/submission-guidelines#loc-laboratory-protocols. Additionally, PLOS ONE offers an option for publishing peer-reviewed Lab Protocol articles, which describe protocols hosted on protocols.io. Read more information on sharing protocols at https://plos.org/protocols?utm_medium=editorial-email&utm_source=authorletters&utm_campaign=protocols.

As the corresponding author, your ORCID iD is verified in the submission system and will appear in the published article. PLOS supports the use of ORCID, and we encourage all coauthors to register for an ORCID iD and use it as well. Please encourage your coauthors to verify their ORCID iD within the submission system before final acceptance, as unverified ORCID iDs will not appear in the published article. *Only*   the individual author can complete the verification step; PLOS staff *cannot*   verify ORCID iDs on behalf of authors.

We look forward to receiving your revised manuscript.

Kind regards,

Yu A. An, M.D., Ph.D.

Academic Editor

PLOS One

Journal Requirements:

If the reviewer comments include a recommendation to cite specific previously published works, please review and evaluate these publications to determine whether they are relevant and should be cited. There is no requirement to cite these works unless the editor has indicated otherwise.

Please review your reference list to ensure that it is complete and correct. If you have cited papers that have been retracted, please include the rationale for doing so in the manuscript text, or remove these references and replace them with relevant current references. Any changes to the reference list should be mentioned in the rebuttal letter that accompanies your revised manuscript. If you need to cite a retracted article, indicate the article’s retracted status in the References list and also include a citation and full reference for the retraction notice.

[Note: HTML markup is below. Please do not edit.]

Reviewers' comments:

Reviewer's Responses to Questions

**Comments to the Author**

1. If the authors have adequately addressed your comments raised in a previous round of review and you feel that this manuscript is now acceptable for publication, you may indicate that here to bypass the “Comments to the Author” section, enter your conflict of interest statement in the “Confidential to Editor” section, and submit your "Accept" recommendation.

Reviewer #1: All comments have been addressed

Reviewer #2: All comments have been addressed

**********

2. Is the manuscript technically sound, and do the data support the conclusions?

The manuscript must describe a technically sound piece of scientific research with data that supports the conclusions. Experiments must have been conducted rigorously, with appropriate controls, replication, and sample sizes. The conclusions must be drawn appropriately based on the data presented.

Reviewer #1: Yes

Reviewer #2: Yes

**********

3. Has the statistical analysis been performed appropriately and rigorously?

Reviewer #1: Yes

Reviewer #2: Yes

**********

4. Have the authors made all data underlying the findings in their manuscript fully available?

The PLOS Data policy requires authors to make all data underlying the findings described in their manuscript fully available without restriction, with rare exception (please refer to the Data Availability Statement in the manuscript PDF file). The data should be provided as part of the manuscript or its supporting information, or deposited to a public repository. For example, in addition to summary statistics, the data points behind means, medians and variance measures should be available. If there are restrictions on publicly sharing data—e.g. participant privacy or use of data from a third party—those must be specified.

Reviewer #1: Yes

Reviewer #2: Yes

**********

5. Is the manuscript presented in an intelligible fashion and written in standard English?

PLOS ONE does not copyedit accepted manuscripts, so the language in submitted articles must be clear, correct, and unambiguous. Any typographical or grammatical errors should be corrected at revision, so please note any specific errors here.

Reviewer #1: Yes

Reviewer #2: Yes

**********

6. Review Comments to the Author

Please use the space provided to explain your answers to the questions above. You may also include additional comments for the author, including concerns about dual publication, research ethics, or publication ethics. (Please upload your review as an attachment if it exceeds 20,000 characters)

Reviewer #1: The manuscript presents a novel and clinically relevant study on mesothelial cell-derived EVs and ANG2-mediated angiogenesis. The revised version has adequately addressed the major reviewer concerns, including mechanistic clarification, additional ANG2 experiments, and improved figure quality. The combined in vitro and in vivo findings strengthen the translational significance of the study. Although direct receptor-binding assays were not performed, this limitation has been appropriately acknowledged and discussed.

Minor revisions including final language polishing, simplification of figure legends, and consistency in statistical reporting are recommended.

The revised manuscript has improved significantly and is suitable for publication after minor editorial revision.

Reviewer #2: (No Response)

**********

7. PLOS authors have the option to publish the peer review history of their article (what does this mean?). If published, this will include your full peer review and any attached files.

If you choose “no”, your identity will remain anonymous but your review may still be made public.

**Do you want your identity to be public for this peer review?**   For information about this choice, including consent withdrawal, please see our Privacy Policy.

Reviewer #1: **Yes:**  DIBYASHREE CHHETRI

Reviewer #2: No

**********

[NOTE: If reviewer comments were submitted as an attachment file, they will be attached to this email and accessible via the submission site. Please log into your account, locate the manuscript record, and check for the action link "View Attachments". If this link does not appear, there are no attachment files.]

To ensure your figures meet our technical requirements, please review our figure guidelines: https://journals.plos.org/plosone/s/figures

You may also use PLOS’s free figure tool, NAAS, to help you prepare publication quality figures: https://journals.plos.org/plosone/s/figures#loc-tools-for-figure-preparation.

NAAS will assess whether your figures meet our technical requirements by comparing each figure against our figure specifications.

# Author comment: Author response to Decision Letter 2

**DOI:** 10.1371/journal.pone.0353115.r005

8 Jun 2026

Reviewer Comment: Minor revisions including final language polishing, simplification of figure legends, and consistency in statistical reporting are recommended.

Response: We thank the reviewer for these suggestions. We have carefully revised the manuscript, accordingly, including additional language polishing, simplification of figure legends, and harmonization of statistical reporting throughout the text, tables, and figures.

Attachment Submitted filename: Response letter-Plos ONE-8.6.26.docx

# Editor report: Decision Letter 2

**DOI:** 10.1371/journal.pone.0353115.r006

**Authors:** Yu An, Yu An, Yu An

19 Jun 2026

Mesothelial cells derived extracellular vesicles promote angiogenesis through the transfer of angiopoietin-2

PONE-D-25-48703R2

Dear Dr. Loewenstein,

We’re pleased to inform you that your manuscript has been judged scientifically suitable for publication and will be formally accepted for publication once it meets all outstanding technical requirements.

Within one week, you’ll receive an e-mail detailing the required amendments. When these have been addressed, you’ll receive a formal acceptance letter and your manuscript will be scheduled for publication.

An invoice will be generated when your article is formally accepted. Please note, if your institution has a publishing partnership with PLOS and your article meets the relevant criteria, all or part of your publication costs will be covered. Please make sure your user information is up-to-date by logging into Editorial Manager at Editorial Manager® and clicking the ‘Update My Information' link at the top of the page. For questions related to billing, please contact billing support.

If your institution or institutions have a press office, please notify them about your upcoming paper to help maximize its impact. If they’ll be preparing press materials, please inform our press team as soon as possible -- no later than 48 hours after receiving the formal acceptance. Your manuscript will remain under strict press embargo until 2 pm Eastern Time on the date of publication. For more information, please contact onepress@plos.org.

Kind regards,

Yu A. An, M.D., Ph.D.

Academic Editor

PLOS One

# Editor report: Acceptance letter

**DOI:** 10.1371/journal.pone.0353115.r007

**Authors:** Yu An, Yu An, Yu An

PONE-D-25-48703R2

PLOS One

Dear Dr. Loewenstein,

I'm pleased to inform you that your manuscript has been deemed suitable for publication in PLOS One. Congratulations! Your manuscript is now being handed over to our production team.

At this stage, our production department will prepare your paper for publication. This includes ensuring the following:

* All references, tables, and figures are properly cited

* All relevant supporting information is included in the manuscript submission,

* There are no issues that prevent the paper from being properly typeset

You will receive further instructions from the production team, including instructions on how to review your proof when it is ready. Please keep in mind that we are working through a large volume of accepted articles, so please give us a few days to review your paper and let you know the next and final steps.

Lastly, if your institution or institutions have a press office, please let them know about your upcoming paper now to help maximize its impact. If they'll be preparing press materials, please inform our press team within the next 48 hours. Your manuscript will remain under strict press embargo until 2 pm Eastern Time on the date of publication. For more information, please contact onepress@plos.org.

You will receive an invoice from PLOS for your publication fee after your manuscript has reached the completed accept phase. If you receive an email requesting payment before acceptance or for any other service, this may be a phishing scheme. Learn how to identify phishing emails and protect your accounts at https://explore.plos.org/phishing.

If we can help with anything else, please email us at customercare@plos.org.

Thank you for submitting your work to PLOS ONE and supporting open access.

Kind regards,

PLOS ONE Editorial Office Staff

on behalf of

Dr. Yu A. An

Academic Editor

PLOS One
